# Ranking of antiseizure medications in a panel of focal seizure models predicts their comparative efficacy in clinical add‐on trials in drug‐resistant focal epilepsy

**DOI:** 10.1002/epi.70210

**Published:** 2026-03-28

**Authors:** Wolfgang Löscher, Pavel Klein

**Affiliations:** ^1^ Translational Epilepsy Research Group, NIFE, Department of Experimental Otology of the ENT Clinics Hannover Medical School Hannover Germany; ^2^ Mid‐Atlantic Epilepsy and Sleep Center Bethesda Maryland USA

**Keywords:** animal models, drug resistance, focal‐onset seizures, kindling, pilocarpine, predictive validity

## Abstract

**Objective:**

Most antiseizure medications (ASMs) have been discovered by testing in animal models, which are generally thought to predict antiseizure activity in patients. However, it is not known whether any of these models (or a combination of models) can predict whether a novel ASM exhibits higher clinical efficacy in focal drug‐resistant epilepsy (DRE) than benchmark ASMs such as carbamazepine or levetiracetam. This important question has plagued epilepsy drug discovery for over three decades and serves as the basis of the present analysis.

**Methods:**

The present analysis includes 23 ASMs that the U.S. Food and Drug Administration (FDA) approved for the treatment of focal‐onset seizures. For assessing comparative preclinical activity, we used the median effective dose (ED_50_) determined in six rodent models of induced generalized or focal‐onset seizures. For assessing comparative clinical efficacy, seizure‐freedom rates recorded during add‐on treatment in randomized controlled clinical trials in patients with focal DRE were used. The preclinical ED_50s_ were ranked from most potent to least potent by SUCRA (*surface under the cumulative ranking curve*). Correlation analysis of preclinical and clinical data was used to determine the predictive validity of the animal models.

**Results:**

Except for the pilocarpine model, none of the seizure models predicted the comparative efficacy of add‐on treatment with ASMs in focal DRE. However, when we combined the ED_50_‐based ranks from two to four models of focal‐onset seizures, highly significant positive correlations with seizure‐freedom rates in the clinical trials were obtained.

**Significance:**

The present study shows that ranking of ASMs across a panel of mechanistically complementary but agnostic models of focal‐onset seizures yields greater translational accuracy and clinical predictivity for patients with focal DRE than rankings based on any single model. This novel finding can be used when defining a path forward that best identifies the most promising investigational compounds to advance to more expensive and time‐consuming clinical investigations.

**Key points**

- The predictive validity of rodent seizure models is incompletely understood.
- We present a pragmatic approach by ranking the potencies of antiseizure medications in six rodent models with induced seizures.
- When these ranks were combined from two to four models of induced focal‐onset seizures, highly significant correlations with clinical efficacy were found.
- Data from models with spontaneous seizures were less predictive, most likely as a result of limitations in the spontaneous seizure model literature.
- The novel approach described here will help to identify the most promising investigational compounds to advance to clinical trials.

## 1 INTRODUCTION

The goal of epilepsy treatment is seizure freedom without adverse effects.^1^ However, despite the availability of more than 30 antiseizure medications (ASMs), one‐third of patients have drug‐resistant epilepsy (DRE), which is associated with increased risks of premature death, injuries, psychiatric and cognitive comorbidity, psychosocial dysfunction, and a reduced quality of life.^2^ Drug resistance is particularly high in focal epilepsies, which explains why most novel ASMs have been developed for focal DRE.^3^, ^4^, ^5^, ^6^ Most ASMs have been discovered by testing in animal seizure models, which are generally thought to have a high predictive validity.^7^, ^8^ Standard models include the maximal electroshock seizure (MES) test of generalized convulsive seizures, the 6‐Hz model of focal seizures, and different types of kindling models of focal‐onset seizures.^7^ Focal seizures in rodent models are typically more resistant to ASMs than models of generalized seizures, such as the MES test, which parallels the clinical responsiveness of focal vs generalized seizures.^9^, ^10^

To facilitate the discovery and characterization of novel ASMs, the National Institute of Neurological Disorders and Stroke (NINDS)/National Institutes of Health (NIH)–funded Anticonvulsant Screening Program (ASP), which started in 1975 and was renamed Epilepsy Therapy Screening Program (ETSP) in 2015, has provided a preclinical screening service for participants from both industry and academia.^10^, ^11^, ^12^, ^13^, ^14^ The ASP/ETSP has played a critical role in the preclinical evaluation of many of the ASMs that have been approved by the U.S. Food and Drug Administration (FDA) and thus made available for the treatment of seizures. During the 50 successful years of the ASP/ETSP, the animal models in use have changed over time such that new assays have been added to the program and others have been discontinued, in line with the program's evolving mission to address the unmet medical needs in epilepsy.^14^ The current testing scheme for DRE includes rodent models used for identification (e.g., MES test, 6‐Hz test, corneal kindling) and differentiation (e.g., amygdala kindled rat, intrahippocampal kainate model) of potential therapies.

However, it is not known whether any of these models (or a combination of models) is capable of predicting whether a novel ASM exhibits a higher clinical efficacy in focal DRE than benchmark ASMs such as carbamazepine (CBZ) or levetiracetam (LEV). This important question has plagued epilepsy drug discovery for over three decades and serves as the basis of the present analysis. Many drugs fail in clinical development because of insufficient efficacy; thus, the predictive validity of preclinical disease models is extremely important in the drug discovery process.^15^, ^16^, ^17^

ASMs approved before 1989 are often regarded as “first generation.” Those developed between 1989 and 2010 are sometimes referred to as “second generation,” whereas those developed subsequently as “third generation.” Although there were 20 second‐ and third‐generation ASMs approved between 1989 and 2019, the proportion of patients with DRE during that time remained unchanged.^18^ In 2019, a novel ASM, cenobamate (CNB), was approved by the FDA for the treatment of focal‐onset seizures.^19^ In the key pivotal Phase 2 clinical trial, up to 20% of the patients were seizure‐free during the maintenance phase (when corrected for placebo response).^20^ For comparison, in pivotal randomized controlled trials (RCTs) of other ASMs approved since 1990, seizure freedom for most ASMs was <4%, rarely 5%–7%, and never >8%.^2^ Based on the high efficacy of CNB, the FDA waived a Phase 3 efficacy study, an unprecedented regulatory decision in epilepsy.

It has been criticized that animal models did not differentiate CNB from other seemingly similarly potent investigational compounds, such as padsevonil and carisbamate (CRS; a close structural analog of the alkyl‐carbamate CNB), both of which were highly effective in animal models but failed in clinical trials in focal DRE.^21^, ^22^, ^23^ In the present study, we performed a retrospective analysis of the preclinical and clinical data of ASMs used in the treatment of focal‐onset seizures to evaluate the predictive validity of animal models.

## 2 MATERIALS AND METHODS

### 2.1 Choice of animal models

Six rodent models of induced seizures were chosen for the present analysis of predictive validity of seizure models: the MES test, the audiogenic seizure, and the 6‐Hz psychomotor seizure models in mice, and the amygdala and hippocampal kindling and the pilocarpine (PILO; with/without lithium [Li]) models in rats. Details of these seizure models and the rationale for their inclusion in the present analysis of predictive validity are described in Appendix S1. Of note, CNB and CRS were both tested in these models, thus allowing us to determine whether these models predicted the dichotomy in clinical efficacy between these drugs. Although the MES, 6‐Hz, and PILO models are acute seizure models in healthy (non‐epileptic) rodents, audiogenic seizure susceptible (e.g., DBA/2) mice carry a chronic genetic reflex epilepsy, and the two kindling models are considered chronic models of temporal lobe epilepsy (TLE; see Appendix S1). In addition to the seizure models, data on “minimal neurotoxicity” from the rotarod test in mice and rats were included in the analysis. Details are described in Appendix S1.

In addition, for comparison with data obtained in the six models with induced seizures, we analyzed data from rodent epilepsy models with spontaneous recurrent seizures (SRS): (i) the intrahippocampal kainate (IHK) mouse model of mesial TLE with highly frequent electrographic hippocampal paroxysmal discharges (HPDs), which allows acute (single‐dose) testing of ASMs; (ii) the intra‐amygdala kainate (IAK) mouse model of TLE with less frequent electroclinical SRS; (iii) a rat model of TLE in which SRS develop after induction of status epilepticus (SE) by i.p. administration of kainate; and (iv) the rat PILO model of post‐SE TLE with SRS (for details see Löscher and White^9^). However, compared to the six models with induced seizures described, fewer data were available for the models with SRS, which limited correlation with clinical data. Furthermore, except for the IHK model, rodent models with infrequent SRS necessitate prolonged administration of ASMs and continuous (24/7) video‐EEG monitoring, so often ASMs are tested only at one dose level, further limiting correlation with clinical data.

### 2.2 Preclinical ASM response

The present analysis includes 23 ASMs that the FDA approved for the treatment of focal‐onset seizures. Two additional FDA‐approved drugs—clorazepate and ethotoin—were not included because these ASMs have not been evaluated in several of the preclinical models used here. Preclinical ASM responses in the six models with induced seizures used for the present analysis were searched in PubMed, Google Scholar, the NINDS PANAChE database (http://panache.ninds.nih.gov; a searchable repository for non‐confidential efficacy data on compounds tested by the ASP/ETSP program^12^), and by data mining, using the principles described by Leenaars et al.^24^ Search terms for preclinical ASM response included the candidate ASM, seizure model, and species. Where more than one reference was available, each complete reference was examined, and a representative reference was selected. In each reference, it was checked whether the methodology used to determine the ASM effect was adequate and reported with sufficient detail (see Appendix S1).

For assessing the comparative preclinical effect of the ASMs, we used the median effective dose (ED_50_) as determined by dose–effect curves. In each seizure model, ASMs were ranked by their ED_50_ from most potent to least potent by SUCRA (*surface under the cumulative ranking curve*), a metric used to quantify the overall effectiveness of a treatment, with scores from 0 (least effective) to 1 (most effective).^25^ Deterministic SUCRA values were calculated by the R package. Ineffective drugs were assigned a SUCRA rank of 0. Drugs that were not tested (or for which an ED_50_ was not reported) received an intermediate score, that is, using mean substitution as a simple imputation technique.^26^, ^27^, ^28^

For the four models with SRS, the percent reduction of seizure frequency was used as a readout. If available, seizure‐freedom rates were used as an additional readout. Furthermore, for the IHK model, ED_50s_ were calculated based on percent reduction of HPD frequency within a group of mice.^9^

### 2.3 Clinical ASM response

The comparative efficacy of second‐ and third‐generation ASMs in RCTs with add‐on treatment in patients with focal DRE was approached in two ways: (1) Seizure‐freedom rates recorded during the maintenance phase of the RCTs, and (2) data from network meta‐analysis (NMA) of RCTs as reported recently by Zhang et al.^29^ For seizure‐freedom rates, the data presented in several previous reviews^2^, ^30^, ^31^, ^32^ and meta‐analyses^33^ were used. All data reported in the reviews were rechecked against the seizure‐freedom rates in the original clinical studies and, where available, the pooled analyses of the pivotal RCTs.

### 2.4 Correlation analysis

The predictive validity of the ED_50s_ of ASMs included in this study was assessed as described by Scannell et al.^16^ According to the latter study, predictive validity is the degree to which the ordering of measures (ED_50s_) from a decision tool (an animal model) would match, across a population of ASMs, the ordering in terms of clinical utility in patients. As described by Scannell et al.^16^ we operationalized predictive validity as the notional Pearson correlation coefficient between the decision tool output and the relevant measure of clinical utility. A positive predictive value was obtained when the correlation coefficient was significant. Correlation analyses were performed by GraphPad Prism 10 (Boston, MA).

For models with SRS, percent reduction of seizure frequency and, if available, seizure freedom rates were used for correlation analyses.

## 3 RESULTS

### 3.1 Comparative potency and efficacy of ASMs in the six preclinical models with induced seizures

Table 1 shows the ED_50s_ of 23 first‐, second‐, and third‐generation ASMs in the six preclinical rodent models with induced seizures used for the present analysis. Only ASMs that are clinically effective against focal and generalized convulsive seizures are illustrated here. If available, data from the ASP/ETSP were preferred because they were blindly determined by experienced researchers in the same laboratory at the University of Utah,^10^, ^14^, ^34^ thereby minimizing the bias of inter‐lab variation.

**TABLE 1:** Preclinical antiseizure potencies of 23 antiseizure medications (ASMs) that are (or were) FDA‐approved for treatment of focal‐onset seizures.

Drug	ED_50_ (mg/kg, i.p.) in seizure models in mice			ED_50_ (mg/kg, i.p.) in focal seizure models in rats					TD_50_ (mg/kg, i.p.) in the rotarod test in mice	TD_50_ (mg/kg, i.p.) in the rotarod test in rats
	MES (50 mA) test of generalized tonic‐clonic seizures	Audiogenic seizure model of generalized convulsive seizures^a^	6‐Hz (44 mA) model of focal seizures in CF‐1 mice^b^	Hippocampal kindling (focal onset, sec. gen. seizures); suprathreshold stimulation (e.g., 400–500 μA)		Amygdala kindling (focal onset, sec. gen. seizures); suprathreshold stimulation (e.g., 400–500 μA)		Lithium‐pilocarpine (or pilocarpine) induced focal and convulsive seizures and SE		
				Focal	Gen.	Focal	Gen.			
**First‐generation ASMs**										
Phenytoin	6.7	2.5	64	121	34	50^c^	30^c^	NE	51	47.1
Carbamazepine	7.8	4.4	25.9	33	4.6	15	8^c^	28.9 (Li‐PILO)	45.4	33.7
Phenobarbital	11.3	3.4	35.3	63	6.7	44	16	15.1 (Li‐PILO)	45.5	41.1
Primidone	11.4	1.3 (running seizures)	NT	NE	45	NE	NE	NT	680	>40
Valproate	263	43	210	255	110	220	190	383 (Li‐PILO)	398	314
**Second‐ and third‐generation ASMs**										
*1. SV2A modulators*										
Levetiracetam	NE	9.8	1089	352^c^		33	32	7.1 (mice; PILO)	2223	1960
Brivaracetam	113	2.4	4.4 (NMRI mice)	.2^d^		44		NE (mice; PILO)	195	370
Oxcarbazepine	14.9	4.2	9.1 (NMRI mice)	NT		>7.5		Effective (100 μM) after hippocampal perfusion (PILO)	110	>500 (p.o.)
Eslicarbazepine acetate	23	NT	77.6	NT		~300 (at ADT)		NT	100	NT
Lamotrigine	5.4	3.5	43.7	27		~40 (at ADT)		NT	30	37.4
Lacosamide	4.5	.63	15.2	13.5		7	12	NE (mice; PILO)	26.8	25.8
**3. Calcium channel modulators**										
Gabapentin	NE	20.3	NE	NE		~30 (at ADT)		NE (Li‐PILO)	>500	91
Pregabalin	11.6	2.7	31.7 (CD1 mice)	20^d^	10^d^	NT		<15 mg/kg (i.v.) (PILO)	144	~100 (p.o.)
**4. GABAergic drugs**										
Vigabatrin^e^	>2000	542	>250 (32 mA; Swiss mice)	NT		~807 (at ADT)		400 (PILO)	1570	NT
Tiagabine	NE	.75	1.0	5.4 (at GST)		4.3 (at GST)		>100 (PILO; mice)	1.29	10.8
**5. Glutamate (AMPA) receptor antagonists**										
Perampanel	1.6 (p.o.)	.47	2.8 (p.o.; ICR mice)	NT		~10 (p.o.)		NT	1.8 (p.o.)	9.14
**6. ASMs with multiple mechanisms**										
Topiramate	18.3	12.1	NE	NE		13.9	10.6	NE (>50) (PILO)	234	299
Zonisamide	41	31.4	111 (32 mA; CD1 mice)	~12 (i.v.; at ADT)		<10 (at GST); NE (>50 i.v.) (at ADT)	<10 (at GST)	NT	105	NT
Rufinamide	15.5	NT	32.9	NT		NE		NT	>500	>350
Cenobamate	9.8	14	16.5	16.4		~4.8		7.0 (Li‐PILO)	58.0	38.9
Carisbamate^f^	7.9	<20 (audiogenic rats)	27.6	22.5		>40		NE (Li‐PILO)	46	39.5
Felbamate	35.5	48.8	241	296		>50		80.5 (Li‐PILO)	220	>500
Retigabine (ezogabine)^g^	9.3	6.8	33	NT		3.2		NT	20.5	10

*Note*: In all of the six models with induced seizures shown here, ASMs were administered before the convulsive stimulus. Note that in the kindling models, only few studies on second‐ and third‐generation ASMs determined ED_50s_ separately for focal vs secondarily generalized convulsive seizures (for Discussion see Appendix S1). References are shown in Table S1.

Abbreviations: ADT, afterdischarge threshold; AMPA, α‐amino‐3‐hydroxy‐5‐methyl‐4‐isoxazolepropionic acid; CC_97_, convulsant current in 97% of mice; ED, effective dose; GST, generalized seizure threshold; Li‐PILO, lithium‐pilocarpine; MES, maximal electroshock seizure; NE, not effective (i.e., <50% protection at highest dose tested or ED_50_ ≥ TD_50_); NT, not tested (or data not found in the public domain); PILO, pilocarpine; SE, status epilepticus; SV2A, synaptic vesicle glycoprotein 2A; TD, toxic dose.

^a^ In audiogenic‐seizure prone DBA/2 or Frings mice; ED_50s_ are shown for clonic seizures.

^b^ Potency of antiseizure medications in the 6 Hz (44 mA) model markedly varies with mouse strain and stimulator used (see Appendix S1); if not indicated otherwise, all data shown here are from CF‐1 mice (Charles River).

^c^ ~50% protection at this dose, but no increase in protection by increasing the dose.

^d^ Minimally active dose (mg/kg); ED_50_ not determined or provided.

^e^ Not quite effective after single doses; effect markedly increases by repeated dosing because of irreversible inhibition of GABA‐T.

^f^ Received provisional FDA‐approval in 2008, but this was withdrawn in 2010 because of inconsistent efficacy across different clinical trials in patients with drug‐resistant focal epilepsy.^22^

^g^ FDA‐approved but withdrawn in 2017.^22^

As described, CNB and CRS were evaluated in all six models. Although CNB was quite effective in all models, CRS failed to suppress seizures induced by Li‐PILO. Furthermore, in amygdala‐kindled rats, in which CRS was tested at 25 and 40 mg/kg, the average seizure score was reduced from 5 to 4.7 and 2.7, respectively,^12^ indicating that the ED_50_ in this model is above the median neurotoxic dose (TD_50_) in the rotarod test (Table 1). In contrast, CNB dose‐dependently decreased both generalized and focal seizures in this model with an ED_50_ of about 5 mg/kg.^35^

In mice, 19 of the 23 ASMs blocked MES seizures, all tested ASMs blocked audiogenic seizures, but only 15 ASMs blocked 6‐Hz (44 or 32 mA) seizures at doses below their TD_50_ in the rotarod test. In amygdala‐kindled rats, nine ASMs were ineffective at tolerable doses, substantiating the high drug resistance of this model. In hippocampal‐kindled rats, 4 of the 13 ASMs tested were ineffective at tolerable doses. Of interest, in contrast to amygdala‐kindled rats, in which CRS was ineffective, both CNB and CRS were effective in hippocampal kindled rats with ED_50s_ of 16.4 and 22.5 mg/kg, respectively. In the PILO model (used with or without Li), 8 of 15 ASMs tested, including CRS, were ineffective. Thus, this model was the most resistant across the six models of induced seizures included in this present analysis.

### 3.2 Ranking of 23 antiseizure medications in six animal models with induced seizures

**FIGURE 1:**
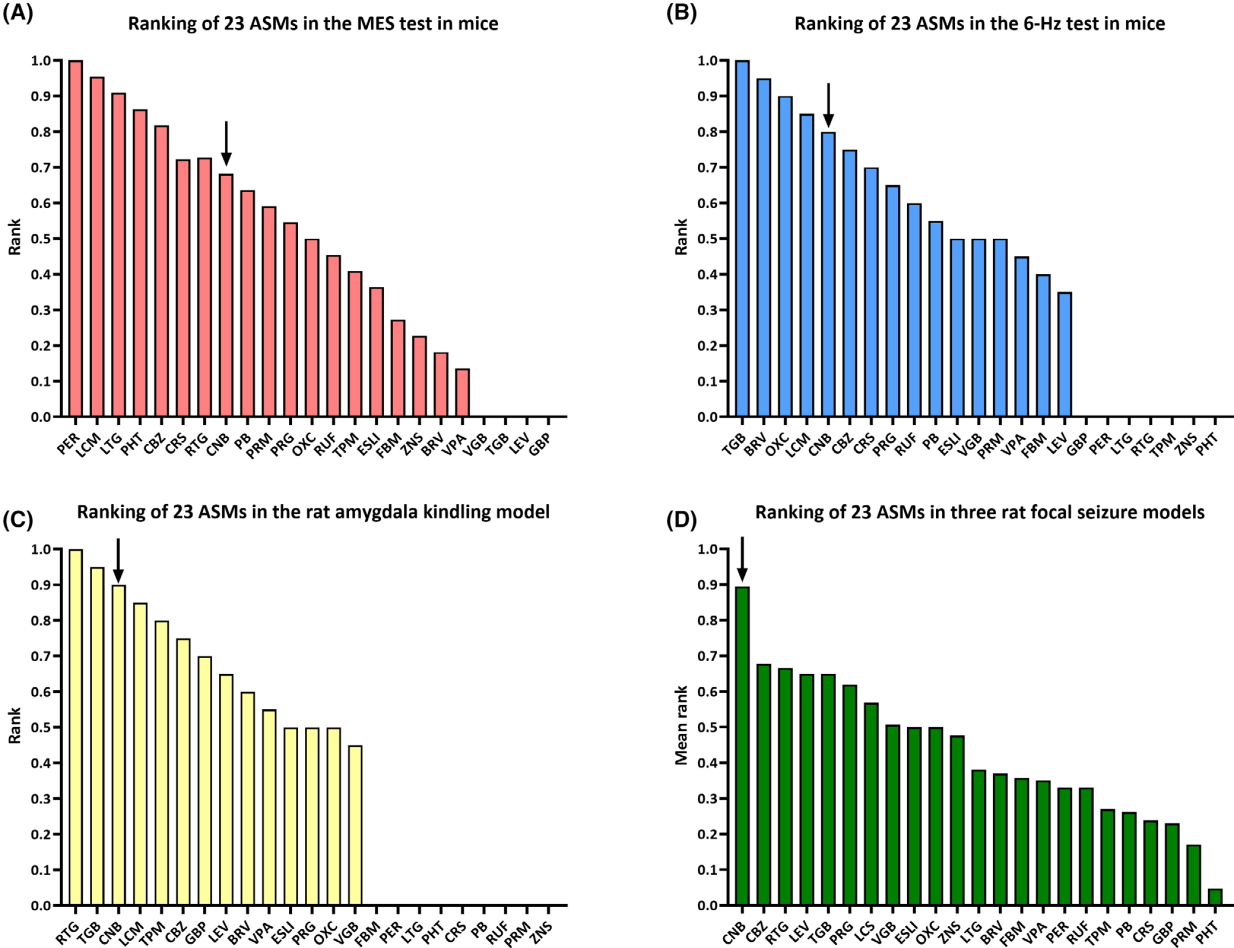
Ranking of 23 first‐, second‐, and third‐generation (ASMs) in rodent seizure models. The rank of cenobamate is indicated by an arrow. Ranking was based on ED_50s_ and performed by SUCRA (see Methods). (A) Ranking in the MES test of generalized convulsive seizures in mice. (B) Ranking in the 6‐Hz test of focal seizures in mice. (C) Ranking in the amygdala kindling model of focal and generalized convulsive seizures in rats. (D) Combined ranking in a panel of three rat models of focal‐onset seizures: the pilocarpine (with/without lithium) model and the hippocampal‐ and amygdala‐kindling models. See Table 1 for the ED_50s_ that were used for ranking in each model. See text for data from additional analyses. ASM, antiseizure medication; BRV, brivaracetam; CBZ, carbamazepine; CNB, cenobamate; CRS, carisbamate; ED_50_, median effective dose; ESLI, eslicarbazepine acetate; FBM, felbamate; GBP, gabapentin; LCM, lacosamide; LEV, levetiracetam; LTG, lamotrigine; MES, maximal electroshock seizures; OXC, oxcarbazepine; PB, phenobarbital; PER, perampanel; PHT, phenytoin; PRG, pregabalin; PRM, primidone; RTG, retigabine; RUF, rufinamide; SUCRA, surface under the cumulative ranking curve; TGB, tiagabine; TPM, topiramate; VGB, vigabatrin; VPA, valproate; ZNS, zonisamide.

The ED_50s_ determined for the 23 ASMs illustrated in Table 1 were used to rank the drugs in each model and combination of models by SUCRA. As shown in Figure 1A, perampanel was the most potent ASM in the MES test, whereas CNB (marked by an arrow) ranked eighth. Similarly, in audiogenic seizure–susceptible mice, perampanel was the most potent ASM, whereas CNB ranked 18th (not illustrated). In the 6‐Hz test, tiagabine was the most potent ASM, whereas CNB had Rank 5 (Figure 1B). In amygdala‐kindled rats, retigabine was the most potent ASM, whereas CNB ranked third (Figure 1C). In hippocampal‐kindled rats, tiagabine was the most potent ASM, whereas CNB ranked fourth(not illustrated). In the PILO model, CNB was the most potent ASM (not illustrated). When the ranks of the three rat models of focal‐onset seizures were summed, CNB had the highest rank, that is, it was the most potent of the ASMs across the three rat focal seizure models (Figure 1D). CRS ranked 20th and was, thus, clearly differentiated from CNB. When the ranks of the 6‐Hz mouse were combined with the ranks of the three rat models of focal‐onset seizures, CNB was again the most effective ASM (not illustrated).

### 3.3 Comparative efficacy of ASMs in clinical trials

Table 2 shows the seizure‐free responder rates (corrected for placebo response) during the maintenance period in Phase 3 or Phase 2 double‐blind, randomized, placebo‐controlled add‐on studies of 16 second‐ and third‐generation ASMs. Seizure‐free responder rates range from 0% (CRS) to 20% (CNB). These responder rates were used to prove the predictive validity of animal models (see Section 3.4).

**TABLE 2:** Indirect comparison of seizure freedom rates of 16 second‐ and third‐generation antiseizure medications in randomized, double‐blind controlled add‐on trials in patients with drug‐resistant focal seizures.

ASM	Percent seizure freedom			Risk ratio (with 95% confidence interval) for 100% responder rate^29^
	Max dose (mg/day)	Percent seizure free (− placebo)	References	
Brivaracetam	200	3.5^a^	[2, 36]	11.0 (3.0, 73.0)
Cenobamate	400	20	[2, 20]	29.0 (5.2, 790.0)
Carisbamate	400	0	[2]	n.d.
Eslicarbazepine acetate	1200	.8	[2, 37]	2.7 (1.5, 5.5)
Gabapentin	1800	.8	[30]	13.0 (2.2, 360.0)
Lacosamide	600	3.9	[2, 38]	6.0 (2.2, 24.0)
Lamotrigine	1800	1.6	[30]	3.8 (1.7, 10.0)
Levetiracetam	3000	7.3	[32]	6.4 (3.4, 14.0)
Oxcarbazepine	2400	2.6	[31]	3.9 (1.6, 11.0)
Perampanel	12	3.0	[2, 39]	4.4 (1.7, 15.0)
Pregabalin	600	1.5	[30]	12.0 (1.9, 330.0)
Retigabine (ezogabine)	1200	4.4	[40]	3.2 (.72, 25.0)
Rufinamide	3200	3.26	[33]	2.0 (.54, 11.0)
Topiramate	1000	5.4	[30]	3.7 (1.7, 9.1)
Vigabatrin	3000	6.7	[32]	14.0 (2.3. 530)
Zonisamide	400	1.4	[30]	2.8 (1.1, 8.7)

*Note*: Where available, seizure‐freedom rates (obtained at the highest daily dose) were taken from the maintenance (12–13 weeks) phase of pivotal trials (lacosamide, eslicarbazepine, retigabine, perampanel, brivaracetam, cenobamate). If more than one study was available, data are from pooled analyses (lacosamide, eslicarbazepine, perampanel, brivaracetam, cenobamate). In addition to seizure‐freedom rates, risk ratios for 100% responder rates (compared with placebo) are shown, obtained from a network meta‐analysis of 75 randomized, double‐blinded, controlled, parallel group, add‐on studies.[^29^] For the latter analysis, the events and number of patients in different dose groups were pooled for studies involving multiple arms with different doses. For 100% responder rate, except for retigabine and rufinamide, all other ASM treatments were significant compared with placebo.

Abbreviations: ASM, antiseizure medication; n.d., not determined.

^a^ Seizure freedom was greater, 4.6% compared with placebo, at a lower dose of 100 mg/d.

NMA is another approach to assess the comparative efficacy (and tolerability) of add‐on treatment with second‐ and third‐generation ASMs in focal DRE.^29^, ^41^ As shown in Table 2, for 100% responder rates, 13 of the 15 ASMs evaluated were significantly different from placebo.^29^ Again, CNB was the most effective drug. The risk ratios calculated for 100% responder rates by Zhang et al.^29^ were used for comparison with efficacy ranks obtained for the animal models, as described in Section 3.4.

### 3.4 An analysis of the predictive value of preclinical data on second‐ and third‐generation ASMs

**FIGURE 2:**
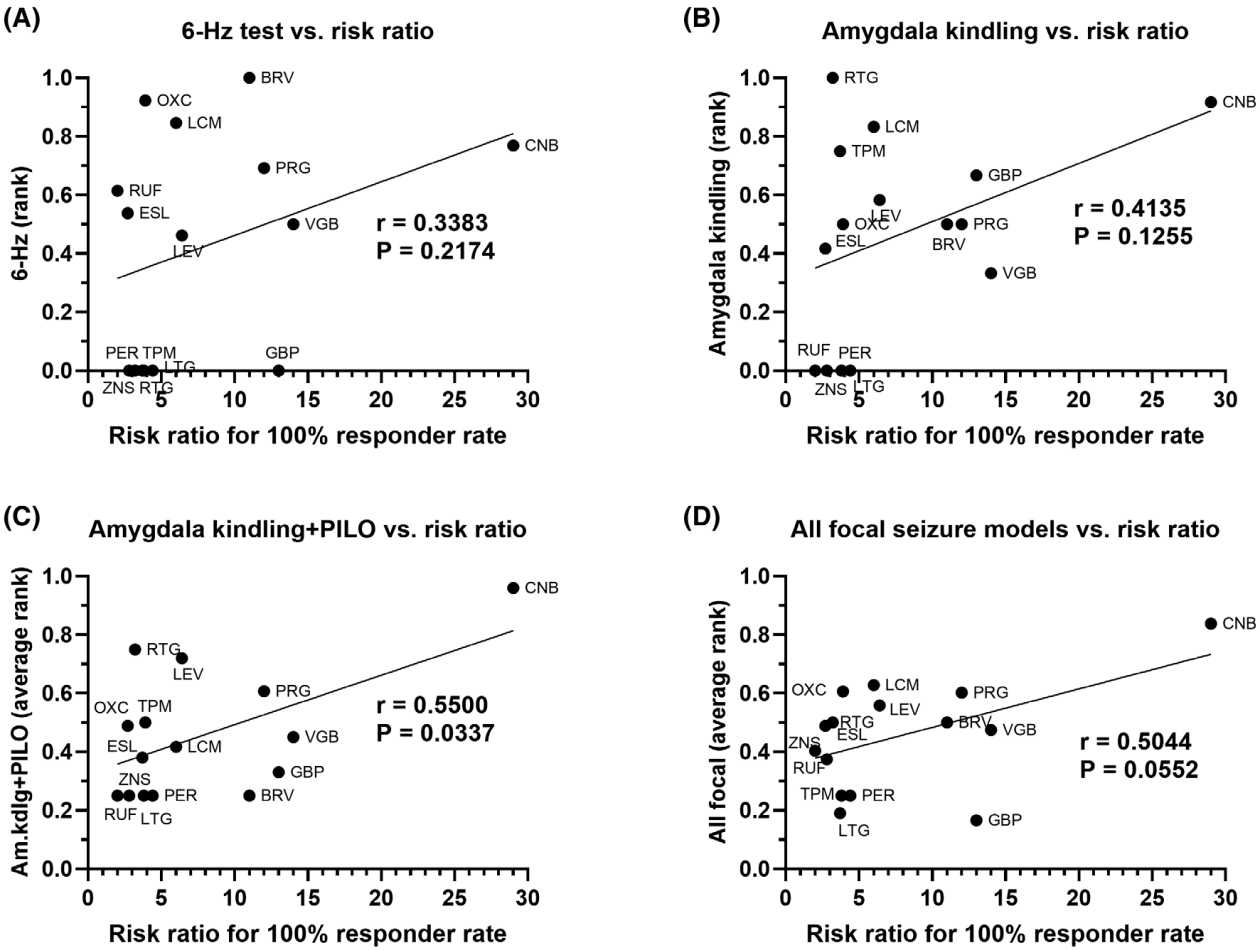
Correlation analyses between the ranks of 15 second‐ and third‐generation ASMs in seizure models and the RR for 100% responder rates determined by network meta‐analysis of randomized controlled add‐on trials by Zhang et al.^29^ Preclinical ranking was based on ED_50s_ and performed by SUCRA (see Methods). The correlation coefficient *r* and the *p* value of *r* were determined by the method of Pearson and are indicated in each graph. (A) Correlation between ranks in the 6‐Hz mouse model and clinical efficacy. (B) Correlation between ranks in the amygdala‐kindling rat model and clinical efficacy. (C) Correlation between combined ranks in the amygdala‐kindling and pilocarpine rat models of focal‐onset seizures and clinical efficacy. (D) Correlation between combined ranks of all four mouse and rat models of focal‐onset seizures (6‐Hz, amygdala kindling, hippocampal kindling, pilocarpine) and clinical efficacy. See text for additional analyses. Am. kdlg., amygdala kindling; ASM, antiseizure medication; BRV, brivaracetam; CNB, cenobamate; ED_50_, median effective dose; ESLI, eslicarbazepine acetate; GBP, gabapentin; LCM, lacosamide; LEV, levetiracetam; LTG, lamotrigine; OXC, oxcarbazepine; PER, perampanel; pilo, pilocarpine; PRG, pregabalin; RR, risk ratio; RTG, retigabine; RUF, rufinamide; SUCRA, surface under the cumulative ranking curve; TPM, topiramate; VGB, vigabatrin; ZNS, zonisamide.

In the NMA reported by Zhang et al.,^29^ risk ratios for 100% responder rates were calculated for 15 second‐ and third‐generation ASMs (Table 2). We ranked these 15 ASMs by ED_50s_ in the six animal models shown in Table 1. As shown in Figure 2, no significant correlations were obtained between the preclinical ranks and clinical risk ratios for the 6‐Hz test and amygdala‐kindling model. A significant correlation was also not obtained for data from the MES test (*r* = −.1228; *p* = .6629), audiogenic mice (*r* = −.2855; *p* = .3024), and the hippocampal kindling (*r* = .0052; *p* = .9851) or PILO (*r* = .3265; *p* = .2350) models (not illustrated). However, a significant correlation was obtained for the combined ranks of the amygdala‐kindling and PILO models (Figure 2C), whereas combined ranks from all four focal seizure models did not significantly correlate with risk ratios (Figure 2D). Similarly, combined ranks of the three rat focal seizure models did not significantly correlate with clinical efficacy (*r* = .4780, *p* = .0715; not illustrated).

**FIGURE 3:**
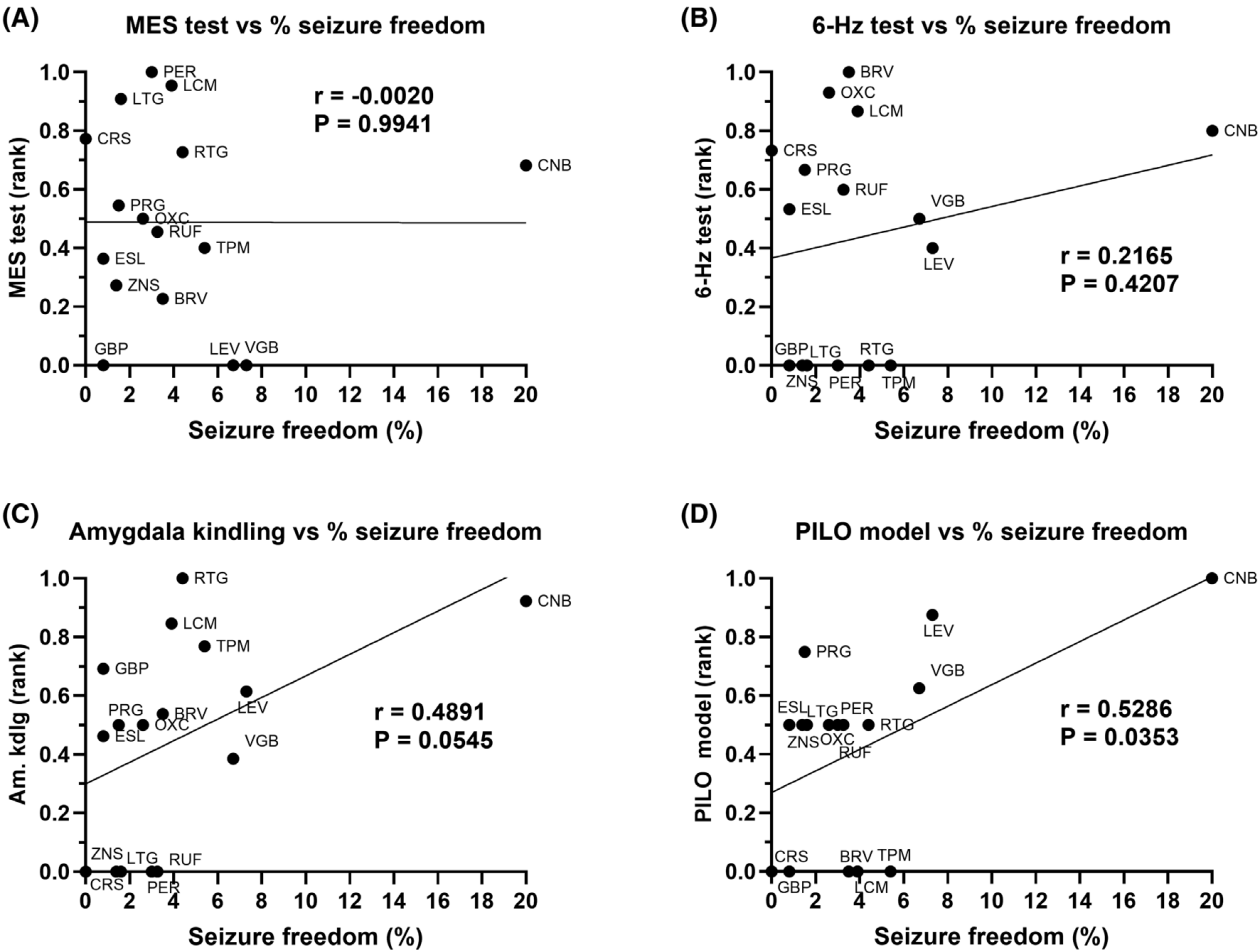
Correlation analyses between the ranks of 16 second‐ and third‐generation ASMs in seizure models and seizure‐freedom rates determined in randomized controlled add‐on trials (see Table 2). Preclinical ranking was based on ED_50s_ and performed by SUCRA (see Methods). The correlation coefficient *r* and the *p* value of *r* were determined by the method of Pearson and are indicated in each graph. (A) Correlation between ranks in the MES test in mice and clinical efficacy. (B) Correlation between ranks in the 6‐Hz mouse model and clinical efficacy. (C) Correlation between ranks in the amygdala‐kindling model and clinical efficacy. (D) Correlation between ranks in the pilocarpine model and clinical efficacy. See text for additional analyses. ASM, antiseizure medication; BRV, brivaracetam; CNB, cenobamate; CRS, carisbamate; ED_50_, median effective dose; ESLI, eslicarbazepine acetate; GBP, gabapentin; kdl, kindling; LCM, lacosamide; LEV, levetiracetam; LTG, lamotrigine; MES, maximal electroshock seizure; OXC, oxcarbazepine; PER, perampanel; pilo, pilocarpine; PRG, pregabalin; RR, risk ratio; RTG, retigabine; RUF, rufinamide; SUCRA, surface under the cumulative ranking curve; TPM, topiramate; VGB, vigabatrin; ZNS, zonisamide.

**FIGURE 4:**
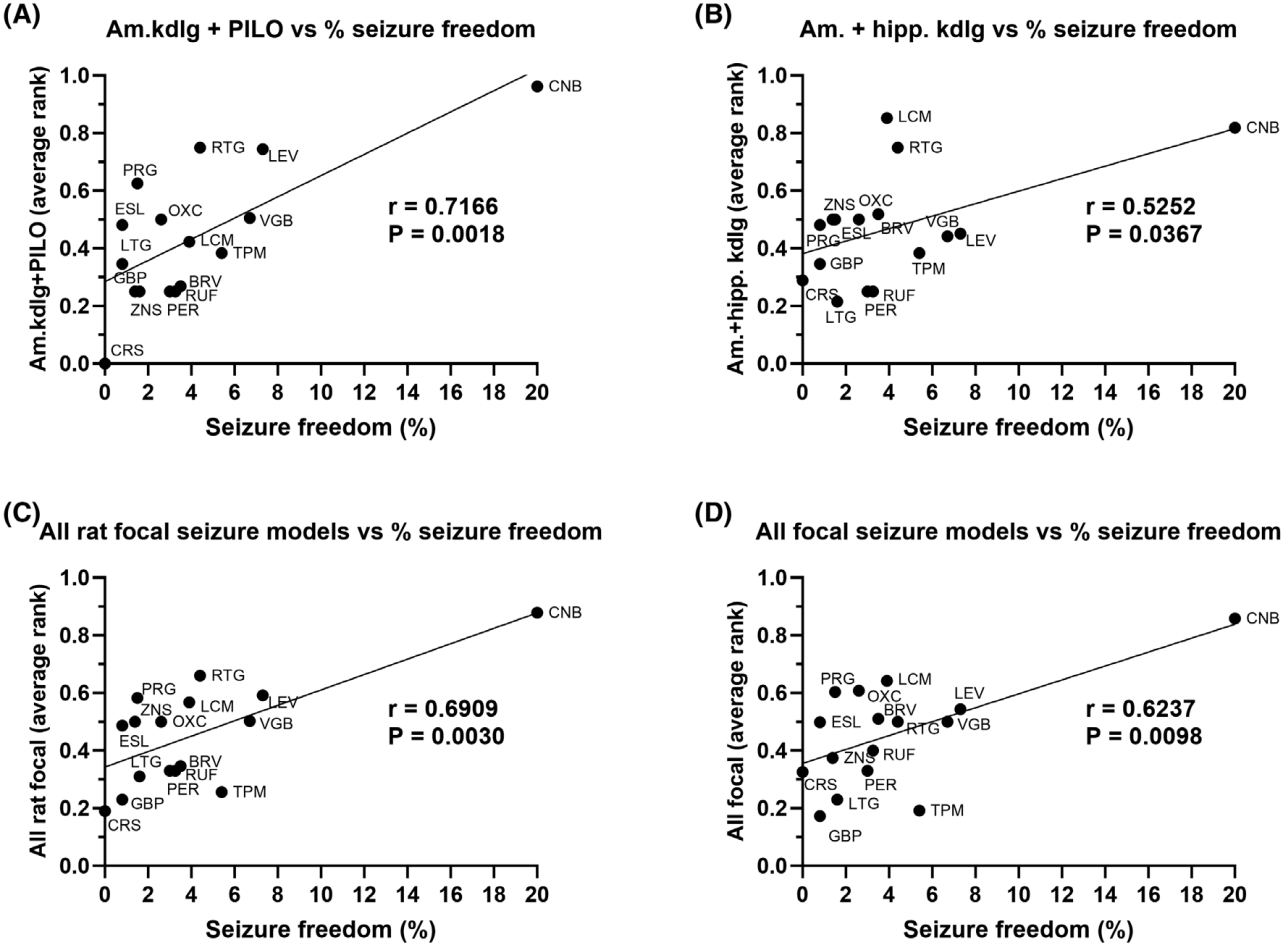
Correlation analyses between the combined ranks of 16 second‐ and third‐generation ASMs in seizure models and seizure‐freedom rates determined in randomized controlled add‐on trials (see Table 2). Preclinical ranking was based on ED_50s_ and performed by SUCRA (see Methods). The correlation coefficient *r* and the *p* value of *r* were determined by the method of Pearson and are indicated in each graph. (A) Correlation between combined ranks in amygdala‐kindling and pilocarpine models in rats and clinical efficacy. (B) Correlation between combined ranks in the amygdala‐ and hippocampal‐kindling rat models and clinical efficacy. (C) Correlation between combined ranks in the three rat models of focal‐onset seizures (hippocampal and amygdala kindling and pilocarpine) and clinical efficacy. (D) Correlation between combined ranks of all four models of focal‐onset seizures (6‐Hz mouse model, hippocampal and amygdala kindling, and pilocarpine) and clinical efficacy. See text for additional analyses. Am. kdlg., amygdala kindling; ASM, antiseizure medication; BRV, brivaracetam; CNB, cenobamate; CRS, carisbamate; ED_50_, median effective dose; ESLI, eslicarbazepine acetate; GBP, gabapentin; LCM, lacosamide; LEV, levetiracetam; LTG, lamotrigine; OXC, oxcarbazepine; PER, perampanel; pilo, pilocarpine; PRG, pregabalin; RR, risk ratio; RTG, retigabine; RUF, rufinamide; SUCRA, surface under the cumulative ranking curve; TPM, topiramate; VGB, vigabatrin; ZNS, zonisamide.

Next, we used the seizure‐freedom data for the 16 ASMs in Table 1 for correlation with animal data, using the same statistical approach as described for the risk ratios. As shown in Figure 3, no significant correlations between preclinical efficacy ranking and clinical efficacy were obtained for the MES test, the 6‐Hz test, or the amygdala‐kindling model. In addition, no significant correlation was obtained when using ED_50s_ of the audiogenic mouse model (*r* = .1536; *p* = .5701) or hippocampal kindling (*r* = .1209; *p* = .6557; not illustrated). In apparent contrast, data from the PILO model correlated significantly with clinical efficacy (Figure 3D). Furthermore, the cumulative ranks of a combination of amygdala kindling and PILO were significantly correlated with seizure‐freedom rates in clinical trials (Figure 4A), whereas the cumulative ranks of a combination of hippocampal kindling and PILO were not (*r* = .4428, *p* = .0859; not illustrated). Significant correlations with clinical efficacy were obtained for the cumulative ranks from a combination of hippocampal and amygdala kindling (Figure 4B), a combination of the three rat models of focal‐onset seizures (Figure 4C), and a combination of all four rodent models of focal‐onset seizures (Figure 4D). Across all analyses, the highest correlation (*r* = .7166, *p* = .0018) was obtained for the combination of the amygdala kindling and PILO models of focal‐onset seizures (Figure 4A).

### 3.5 Comparative potency and efficacy of ASMs in preclinical TLE models with spontaneous seizures

Table 3 shows the effects of ASMs in four chronic rodent models of TLE with SRS: the IHK and IAK mouse models and the systemic (i.p.) kainate and PILO rat models. For 7 of the 23 ASMs evaluated in the induced seizure models (Table 1), including CNB and CRS, no data were found for the SRS models in the public domain. Most data were available for the IHK mouse model and the systemic kainate rat model; so, subsequent correlation analyses were performed for these two models. For the IHK mouse model, both the ED_50_ and the maximal reduction of HPD frequency at high doses of ASMs are shown in Table 3. In the three other models, often only one high dose was tested per ASM, so reduction of SRS frequency was used as a readout. In addition, percent seizure freedom was reported for 16 ASMs, using a 2‐week crossover design in the systemic kainate model of TLE.^42^

**TABLE 3:** Antiseizure effects of antiseizure medications (ASMs) in rodent models with spontaneous recurrent seizures.

Drug	ASM effects in models with SRS in mice			ASM effects in models with SRS in rats			TD_50_ (mg/kg i.p.) in the rotarod test in mice	TD_50_ (mg/kg i.p.) in the rotarod test in rats	References for SRS models (see Table S1 for references for TD_50s_)
	Effect on HPDs following acute treatment with several doses per ASM in the intrahippocampal kainate model		Percent reduction of SRS frequency during prolonged treatment with ASMs in intra‐amygdala kainate model	Effect on SRS during prolonged treatment with ASMs in systemic (i.p.) kainate model (at highest dose tested)		Reduction of SRS frequency during prolonged treatment with ASMs in systemic pilocarpine model			
	Maximal reduction of HPD frequency (dose in mg/kg i.p.)	ED_50_ (mg/kg i.p.) for reduction of HPD frequency		Reduction of seizure frequency	Percent seizure freedom				
**First‐generation ASMs**									
Phenytoin	45% (50)	>50	NE (20 mg/kg bid)	NE (10 mg/kg bid)	NE (10 mg/kg bid)	86% (100 mg/kg/day)	51	47.1	[42, 43, 44, 45]
Carbamazepine	73% (100)	84 (>TD_50_)	NE (30 mg/kg tid)	82% (30 mg/kg tid)	64% (30 mg/kg tid)	95% (120 mg/kg/day)	45.4	33.7	[42, 43, 45, 46]
Phenobarbital	98% (60)	25	67% (25 mg/kg bid); 47% (50 mg/kg bid)	93% (30 mg/kg qd)	83% (30 mg/kg qd); ED_50_ = 12 mg/kg	94% (40 mg/kg/day)	45.5	41.1	[42, 43, 45, 46]
Primidone	NT	NT	NT	NT	NT	NT	680	>40	
Valproate	91% (400)	280	67% (240 mg/kg tid)	63% (200 mg/kg tid)	40% (200 mg/kg tid)	40% (450 mg/kg/day); 90% (600 mg/kg/day)	398	314	[42, 43, 45, 46]
**Second‐ and third‐generation ASMs**									
*1. SV2A modulators*									
Levetiracetam	95% (800)	580	NE (80 mg/kg/day)	68% (150 mg/kg bid)	NE (150 mg/kg bid)	62% (389 mg/kg/day)	2223	1960	[42, 46, 47, 48]
Brivaracetam	NT	NT	NT	NT	NT	NT	195	370	
*2. Sodium channel modulators*									
Oxcarbazepine	NE (10 and 30)	NE	NT	NT	NT	NT	110	>500 (p.o.)	[49]
Eslicarbazepine acetate	NT	NT	NT	NT	NT	NT	100	NT	
Lamotrigine	47% (90)	>90	NT	NE (30 mg/kg bid)	NE (30 mg/kg bid)	NT	30	37.4	[42, 46]
Lacosamide	NT	NT	NT	NE (30 mg/kg qd)	NE (30 mg/kg qd)	40% (80 mg/kg/day)	26.8	25.8	[42, 50]
*3. Calcium channel modulators*									
Gabapentin	60% (500)	ND	NT	54% (300 mg/kg bid)	47% (300 mg/kg bid)	NT	>500	91	[42]; PANAChE
Pregabalin	66% (100)	40	NT	NT	NT	NT	144	~100 (p.o.)	[46]
*4. GABAergic dru*									
Vigabatrin	91% (100)	52	NT	NT	NT	NT	1570	NT	[46]
Tiagabine	91% (1.2)	.33	NT	60% (8 mg/kg bid)	20% (8 mg/kg bid)	NT	1.29	10.8	[42, 46]
*5. Glutamate (AMPA) receptor antagonists*									
Perampanel	96% (2.0)	.7	NT	71% (1.5 mg/kg tid)	40% (1.5 mg/kg tid)	46% (8 mg/kg/day)^a^	1.8 (p.o.)	9.14	[42, 51, 52]
*6. ASMs with multiple mechanisms*									
Topiramate	16 (300)	ND	NT	97% (300 mg/kg bid)	71% (300 mg/kg bid); ED_50_ = 31.1 mg/kg	NE (10 mg/kg/day)	234	299	[42, 53]; PANAChE
Zonisamide	NT	NT	NT	NT	NT	NT	105	NT	
Rufinamide	NT	NT	NT	NT	NT	NT	>500	>350	
Cenobamate	NT	NT	NT	NT	NT	NT	58.0	38.9	
Carisbamate	NT	NT	NT	NT	NT	NT	46	39.5	
Felbamate	NT	NT	NT	NT	NT	NT	220	>500	
Retigabine (ezogabine)	NE (30)^b^	NE	NT	95% (5 mg/kg tid)	80% (5 mg/kg tid)	NT	20.5	10	[42, 54]

*Note*: ASMs were tested with single doses in the intrahippocampal kainate (IHK) mouse model (which was possible because of the extremely high frequency of electrographic seizures in this model), whereas prolonged drug administration and continuous video‐EEG monitoring were used in the other models, which restricts the number of doses that could be tested. For all models, the reduction of seizure frequency during treatment is shown. Furthermore, percent seizure freedom is shown for the systemic kainate rat model. No significant seizure freedom was obtained in the intra‐amygdala kainate mouse model for the ASMs shown here.^45^ In the IHK model, seizure‐freedom rates were not reported by Duveau et al.^46^ Seizure‐freedom rates were reported by Leite and Cavalheiro^43^ for the pilocarpine model, but these data are not robust because of the lack of continuous (24/7) video‐EEG monitoring. ED_50s_ were calculated for the reduction of HPD frequency in the IHK model^9^ and seizure‐freedom rates during treatment with phenobarbital and topiramate in the systemic kainate rat model.^42^ TD_50s_ following acute treatment are shown for comparison.

Abbreviations: ADT, afterdischarge threshold; AMPA, α‐amino‐3‐hydroxy‐5‐methyl‐4‐isoxazolepropionic acid; bid, two times daily; ED, effective dose; GST, generalized seizure threshold; HPD, hippocampal paroxsmal discharge; NE, not effective; ND, not determined; NT, not tested (or data not found in the public domain); qd, once daily; PANAChE, Public Access to Neuroactive & Anticonvulsant Chemical Evaluations; SE, status epilepticus; SRS, spontaneous recurrent seizures; SV2A, synaptic vesicle glycoprotein 2A; TD, toxic dose; tid, three times daily.

^a^ In responders (7/13 rats).

^b^ In a subset of mice, retigabine reduced HPDs and decreased body temperature.

First, we tested whether the ED_50s_ for reduction of HPD frequency in the IHK model correlate with ED_50s_ determined in the 6‐Hz model, both considered models of difficult‐to‐treat focal seizures. As shown in Figure S5A, when ED_50s_ available for 11 ASMs in both models were ranked by SUCRA, no significant correlation was determined. However, ED_50s_ are differently calculated by dose–response experiments in the two models. ED_50_ in the 6‐Hz model is the dose protecting 50% of mice from focal seizures, whereas the ED_50_ in the IHK model represents the dose reducing HPD frequency by 50% in a group of epileptic mice.^9^

Next, we analyzed whether seizure‐freedom rates determined for ASMs in the systemic kainate model correlate with seizure‐freedom rates obtained in clinical trials. For only six ASMs in Table 2, such data were available for the kainate model, and no significant correlation with clinical efficacy was obtained (Figure S5B).

Similarly, the maximal reduction of SRS in the IHK model did not correlate with clinical seizure‐freedom rates (Figure S5C). When maximal reduction of SRS in the IHK and systemic kainate models was ranked by SUCRA and average SUCRA values for 10 ASMs were correlated with clinical efficacy, again, no significant correlation was obtained (Figure S5D).

## 4 DISCUSSION

To our knowledge, this is the first approach using principles of predictive validity analysis to examine whether preclinical ASM data predict the comparative efficacy of add‐on treatment with novel ASMs in focal DRE. As suggested previously,^55^ the MES test did not predict the comparative efficacy of add‐on treatment with ASMs in focal DRE. This was also the case for the audiogenic seizure model of generalized seizures and, except for the PILO model, rodent models of focal‐onset seizures. In contrast, ranking of ASMs in a panel of models of focal‐onset seizures predicted comparative clinical efficacy. Significant correlations with clinical efficacy were obtained both for seizure freedom rates in add‐on clinical RCTs in focal DRE and for risk ratios for seizure freedom obtained from NMA of such trials.

Furthermore, by combining the ranks of all 23 ASMs in the three rat models of focal‐onset seizures, CNB received the top rank and was clearly separated from CRS, thus paralleling the marked difference in clinical efficacy between these two alkyl‐carbamates. This indicates that anticonvulsant ED_50s_ are a suitable parameter for the comparative ranking of ASMs. As discussed in Appendix S2, depending on the seizure model used, the ED_50_ may reflect both potency and efficacy of an ASM. Indeed, as indicated by the present data, the comparative ranking of ED_50s_ can be used to predict clinical efficacy, particularly when combining data from different focal seizure models.

### 4.1 Evaluation of drug efficacy in clinical trials

During clinical development, novel ASMs for focal epilepsy are evaluated in randomized, placebo‐controlled add‐on trials in patients with focal DRE.^56^, ^57^ Although there is universal agreement that sustained seizure freedom should be the primary objective of ASM treatment, in adjunctive‐therapy trials, typically only few patients achieve this goal, and therefore demonstration of efficacy has to rely on the use of other endpoints such as percent reduction in seizure frequency or responder rate (proportion of patients with at least 50% reduction in seizure frequency compared with baseline).^56^, ^57^ Although the use of the latter endpoints is acceptable for regulatory purposes, it has little clinical relevance. Thus, although it remains unclear whether the seizure‐freedom outcomes in clinical trials in refractory patients are predictive of longer‐term seizure freedom in clinical practice,^32^ they do represent a reference point when assessing new ASMs^31^ and may be used to assess the comparative efficacy of these drugs.^2^ However, although the trial methodology and patient population are fairly comparable for most new ASMs, results from different drug studies are not directly comparable because they were not obtained in a head‐to‐head comparison (see Section 4.5).

### 4.2 Multimodal approaches for assessing predictive validity

Multimodal approaches have previously been shown to improve the prediction of epilepsy development, its diagnosis, prognosis, and response to treatment.^58^, ^59^ Indeed, because of the complexity of epilepsy and its etiologies, combining multiple biomarkers can significantly improve the accuracy of predictions compared to using single biomarkers alone.^58^, ^59^ The present study demonstrates that a multimodal combinatorial approach by ranking ASMs in a panel of diverse, mechanistically complementary but agnostic seizure models can be used to improve the predictive validity of the models. This is an important finding that could be easily used for large ASM discovery programs such as the ETSP. The present finding that combinatorial approaches across a panel of preclinical models yield greater translational accuracy and clinical predictivity than rankings based on any single model is supported by studies of a variety of preclinical data with diverse drugs.^60^

Concerning the differentiation of CNB from CRS, two alkyl‐carbamates with contrasting clinical efficacy in focal DRE,^2^, ^22^ ranking of ED_50s_ in the three rat models of focal‐onset seizures used for the present analysis clearly demonstrated the superiority of CNB. This was particularly related to the lack of efficacy of CRS in the Li‐PILO and amygdala‐kindling models, whereas CRS was almost as effective as CNB in the MES, 6‐Hz, and hippocampal‐kindling models. Thus, in contrast to a previous notion,^21^ this substantiates that a battery of models of difficult‐to‐treat focal‐onset seizures is needed to correctly predict the clinical efficacy of novel ASMs.

### 4.3 An alternative approach for analyzing the predictive validity of seizure models

Anderson et al.^61^ recently described a pragmatic decision tree approach to support efficient resource utilization for novel ASM discovery for focal‐onset seizures in adult patients. The Praxis Analysis of Concordance (PAC) framework was implemented to assess the translational concordance between preclinical and clinical ASM response for 32 FDA‐approved ASMs. The preclinical potency of these ASMs was examined in a total of 23 seizure models across multiple species. Based on this analysis, the preclinical models with the highest predictive validity for focal‐onset seizures were the MES test, the mouse audiogenic seizure model, the 6‐Hz (32 mA) mouse model, and the rat amygdala‐kindling model. However, the analysis included a number of ASMs (e.g., ethosuximide, methsuximide, clonazepam, diazepam, clobazam, ganaxolone, stiripentol, everolimus, fenfluramine, and cannabidiol) that are not approved for focal‐onset seizures in adults and some (ethosuximide, methsuximide, and stiripentol) that are not effective against this seizure type. Furthermore, the MES test and audiogenic seizure‐susceptible mouse, albeit susceptible to numerous ASMs, are models of generalized seizures and thus have little to no face or predictive validity for focal‐onset seizures, as substantiated by our analysis. For instance, both CNB and padsevonil are effective in the MES and audiogenic DBA/2 audiogenic mouse model, but only CNB is effective in patients with focal DRE.^21^, ^22^, ^62^

In the study of Anderson et al.^61^, the preclinical ASM response for each preclinical seizure model was graded based on protective index (PI) values, although, as discussed in Appendix S1, the size of the PI has little predictive relevance. The clinical ASM response used for comparison with the preclinical data was based on perceived efficacy and prescribing patterns in adult patients with focal epilepsies. Despite these potential limitations, the approach described by Anderson et al.^61^ yielded interesting data that may support ongoing research and ASM development efforts.

The present, more pragmatic approach for determining the predictive validity of rodent seizure models was inspired by the methods described by Sams‐Dodd^15^ and Scannell et al.^16^ Our approach differed in several aspects from the approach used by Anderson et al.^61^ In particular, we included only ASMs that are FDA‐approved for the treatment of focal‐onset seizures. Second, except for the MES and audiogenic seizure tests, we only included rodent models of difficult‐to‐treat focal‐onset seizures. Third, for clinical efficacy, we used seizure‐freedom rates from add‐on treatment in RCTs in patients with focal DRE. And fourth, rather than comparing only single models with the clinical efficacy of ASMs, we included a multimodal approach with panels of models in our analysis. Only the latter approach resulted in highly significant correlations with clinical efficacy.

### 4.4 How to explain the apparent lack of predictive validity of chronic rodent models with spontaneous seizures?

Based on the higher resemblance with the human disease, one would expect that determining the efficacy of ASMs in chronic models of TLE with SRS would result in a higher predictive validity than that obtained by ED_50s_ in models with induced seizures. However, Löscher^63^ reported that the pharmacology of elicited kindled seizures in fully amygdala‐kindled rats and SRS in post‐SE models of TLE is remarkably similar, which is in line with the high predictive value of ASM efficacy against focal seizures in the kindling model of TLE.^9^

Surprisingly, the present analysis of ASM efficacy data in four TLE models with SRS failed to result in a significant correlation with clinical data. There may be several reasons for this unexpected result. As discussed in Section 2.1, compared to the six models with induced seizures, fewer data were available for the models with SRS, which limited correlation with clinical data. In the study of Anderson et al.^61^ only one model with SRS, the IHK mouse model, was evaluated. The authors noted that less than half of all approved ASMs have data publicly available for this model, thus resulting in limited data depth.

Furthermore, as shown here, except for the IHK model, ASMs were often tested only at one dose level in models with SRS, further limiting correlation with clinical data. If this dose was not adequately chosen, the model may produce false‐negative data. Except for the IHK model, the prolonged drug testing that is needed in models with infrequent electroclinical SRS is affected by the rapid drug elimination of rodents, again leading to the risk of false‐negative data if the short half‐lives of most drugs in rodents are not adequately addressed in the treatment protocol.^8^ In addition, as in humans with epilepsy, epileptic animals do not homogenously respond to ASMs, resulting in subgroups (responders vs non‐responders) that are often averaged in data analysis.^9^ However, the most relevant issue for the present analysis is that many of the more recent ASMs, including CNB, were not yet evaluated in models with SRS. Thus, we simply do not know at present whether data obtained in models with SRS are more predictive of clinical efficacy than models with induced focal seizures.

### 4.5 Limitations of the present study

Our study has several limitations. Analysis of the hippocampal‐kindled rat and the PILO rat models was limited by the depth of publicly available data. Another potential limitation relates to the heterogeneity across preclinical studies, including, but not restricted to, varying rodent strains, sexes, drug formulations, pretreatment times, and the equipment (e.g., stimulators) used (see Appendix S1 for details).

Another potential limitation is the strategy used to estimate clinical efficacies of ASMs, that is, seizure‐freedom rates obtained during add‐on treatment in RCTs. Because of the paucity of head‐to‐head comparative RCTs between the large number of new ASMs approved since 1989, only indirect comparisons can be made. Several methods have been used in the past. Arguably, the most approximate method is the comparison of efficacy, that is, seizure‐freedom rates during the maintenance phase of pivotal RCTs with a similar duration of the maintenance phase. This method is not ideal, as patients who may drop out during the titration phase because of adverse events related to the forced titration of RCTs may distort efficacy numbers. It is further limited by the fact that studies of different ASMs done across a 30‐year time frame may have included different patient populations, different concomitant ASMs, and sometimes different study designs, and that results of studies done later may be affected by concomitant treatment with previously approved new ASMs. Nevertheless, it provides a reasonable basis for comparing efficacy between ASMs, and although a seizure‐freedom difference of 1%–2% between drugs may not be meaningful, large differences likely accurately differentiate between drugs' efficacies.

Similarly, NMAs of efficacy across ASMs are affected by significant limitations, including a variety of study designs, such as differences in dosing, treatment duration, outcomes, and outcomes by treatment vs maintenance treatment duration. On the other hand, as outlined by Song et al.^64^ adjusted indirect comparisons usually, but not always, agree with the results of head‐to‐head randomized trials. When there is no or insufficient direct evidence from head‐to‐head randomized trials, the adjusted indirect comparison can provide useful and/or supplementary information on the relative efficacy of competing interventions. The validity of the adjusted indirect comparisons depends on the internal validity and similarity of the included trials.

Although we found significant correlations between ranks of ASMs in focal seizure models and clinical efficacy, most correlation coefficients were relatively low. The highest correlation coefficient (*r* = .7166, *p* = .0018) was obtained when comparing the combined ranks of the rat amygdala‐kindling and PILO models with seizure‐freedom rates in RCTs. The combined ranking of rat focal seizure models clearly indicated that CNB is the most effective drug among the 16 second‐ and third‐line ASMs evaluated.

A general limitation of all approaches using preclinical drug potencies for correlation with clinical efficacy is that translation to human efficacy is affected by pharmacokinetics, pharmacodynamics, target biology, and interspecies differences.^65^ However, with these limitations in mind, preclinical models are important decision tools in drug discovery, and potency can be a good preclinical marker of the therapeutic potential of a drug.^15^, ^16^, ^65^

The approach described here should be validated and further refined by data from one lab, such as the lab performing the ETSP at the University of Utah. Although we restricted the choice of animal models with induced seizures to those for which data of CNB and CRS were available, one could envision substituting the hippocampal kindling or PILO models with other chronic models, such as corneal kindling. Furthermore, once more data on novel ASMs such as CNB are available for TLE models with SRS, it would be important to reanalyze the potential concordance between ASM efficacies in such models and clinical efficacy.

For further validation of the present approach, it will be critical to prove whether the approach will identify a treatment that surpasses CNB, which is currently the ASM to beat. Given the many nuances to clinical efficacy, particularly human tolerability and the potential for synergy with the other ASMs a patient may be taking, the answer to this particular question will necessarily have to await clinical trials.

## 5 CONCLUSIONS

The present study shows that ranking of ASMs across a panel of difficult‐to‐treat focal‐onset seizure models yields greater translational accuracy and clinical predictivity in patients with focal DRE than rankings based on any single model. Careful selection of animal models is essential, especially in determining which features of the human disease are accurately replicated in the models, to optimize the selection of disease models more likely to predict human response.^15^, ^66^, ^67^, ^68^ As discussed in Appendix S1 and substantiated by the present data, chronic models of TLE, such as amygdala kindling, obviously resemble the human condition (focal DRE) better than acute models, such as the MES and 6‐Hz tests. Given that the goal of preclinical efficacy testing is to define a path forward that best identifies the most promising compounds to advance to more expensive and time‐consuming clinical investigations, the results of this study clearly support the inclusion of a panel of rodent models of drug‐resistant focal seizures in the early evaluation of an investigational drug for the treatment of epilepsy. Activity in these models would definitely support the advancement of an effective drug for the treatment of drug‐resistant human epilepsy.

## AUTHOR CONTRIBUTIONS

**Wolfgang Löscher:** Conceptualization; investigation; methodology; formal analysis; visualization; writing—original draft; writing—review and editing. **Pavel Klein:** investigation; formal analysis; validation; writing—review and editing. All authors have read and approved the submitted version.

## FUNDING INFORMATION

No funding was received for this review.

## CONFLICT OF INTEREST STATEMENT

Wolfgang. Löscher is a member of the External Consultant Board of the National Institutes of Neurological Disorders and Stroke (NINDS) Epilepsy Therapy Screening Program (ETSP). He is co‐founder and Chief Scientific Officer (CSO) of PrevEp, Inc. (Bethesda, MD). He has received in the past 5 years consultancy fees from Lundbeck, Angelini, Clexio, Selene, Axonis, SynapCell, Sintetica, ND Capital, Atlas Venture, Cogent Biosolutions, Ovid, Idorsia, and Addex. Pavel Klein has served as a consultant, advisory board member or speaker (2020–2025) for Abbott, Angelini Pharma, Aquestive Therapeutics, Arvelle Therapeutics, Aucta Pharmaceuticals, Dr. Reddy's, Eisai, GRIN Therapeutics, Jazz Pharmaceuticals, Longboard Pharmaceuticals, Neurelis, Neurona Therapeutics, Paladin Pharma, SK Life Science, Sunovion, UCB Pharma, UNEEG, UniQure, and Xenon Pharma; is a member of the Data and Safety Monitoring Board for Neurona Therapeutics; is a member of Medical Advisory Board of Stratus and of the Scientific Advisory Boards of OB Pharma and of NEUmirna, is the Chief Executive Officer (CEOf PrevEp, Inc. (Bethesda, MD); and has received research support from CURE/Department of Defense and from the NINDS.

## ETHICS STATEMENT

We confirm that we have read the Journal's position on issues involved in ethical publication and affirm that this report is consistent with those guidelines.

## Supporting information


Appendix S1.

Appendix S2.

Figure S5.

Table S1.

## Data Availability

The data that support the findings of this study are available from the corresponding author upon reasonable request.
