# Supplementary material for: Ranking of antiseizure medications in a panel of focal seizure models predicts their comparative efficacy in clinical add‐on trials in drug‐resistant focal epilepsy
Source: Epilepsia. 2026 Mar 28;67(7):3719–37. doi: 10.1002/epi.70210 (PMC13360943; doi:10.1002/epi.70210)
Supplement: Supplementary file 1 — Appendix S1. [file EPI-67-3719-s004.pdf]

Appendix S1

Animal models with induced seizures used for the analyses

1. The MES mouse model of generalized tonic-clonic seizures

The MES test, which was originally described for rodents by Toman and collaborators about 80 years ago (Toman et al., 1946), is probably the best-validated preclinical test that predicts drugs effective against generalized tonic-clonic seizures (Bialer and White, 2010). In this test, tonic-clonic seizures are induced by transcorneal or, less often, transauricular application of a short (0.2 sec) suprathreshold electrical stimulus in normal mice (50 mA) or rats (150 mA). The stimulus strength is about 5-10 times higher than the individual seizure threshold in mice or rats, so that 100% of the animals display tonic-clonic seizures. The anticonvulsant activity of the drug is determined as a quantal endpoint, that is, the presence or absence of hind limb extension. As shown in Table 1, antiseizure medications (ASMs) with different mechanisms of action are highly effective in this test. It is important to note that, in contrast to most other models discussed here, the MES test is not a model of drug-resistant seizures.

In addition to the use of the MES test as a model of generalized tonic-clonic seizures, it was proposed that this test may also predict ASMs with efficacy against focal seizures (Krall et al., 1978a; Krall et al., 1978b), but the lack of anti-MES activity of several ASMs (e.g., levetiracetam [LEV], tiagabine, vigabatrin) that subsequently were shown to suppress focal seizures in epilepsy patients strongly argues against this idea (Löscher, 2011). Nevertheless, because the MES test is still widely used in ASM discovery, we reexamined the potential value of this test in predicting the efficacy of ASMs to suppress focal seizures in clinical trials in the present study.

Although the MES test is generally thought to be a robust test of antiseizure potency, several technical, biological, and pharmacological factors may affect drug testing in this model (Swinyard, 1972; Löscher et al., 1991; Castel-Branco et al., 2009). The potentially

most important factor is the stimulation device that should deliver either a constant current or constant voltage at a frequency of 50-60 Hz for 0.2 sec. The intrinsic impedance of mice (10-30 kOhm) during stimulation necessitates that the stimulator can deliver a current of 50 mA independent of the impedance of the animal, which is only possible with a powerful self-adjusting stimulus voltage (Löscher and Schmidt, 1988; Castel-Branco et al., 2009). Otherwise, the stimulus delivered will be below 50 mA, which may lead to false positive anticonvulsant potencies in the MES test. The same problem also exists for the 6-Hz model (see below). Most commercial stimulators are unable to deliver 50 mA (mice) or 150 mA (rats) independently of the external resistance of the animal, because the self-adjusting stimulus voltage is not high enough (cf., Löscher et al., 1991; Leclercq and Kaminski, 2015). This obviously affects any inter-laboratory comparison of ASM potencies in the MES model. As a consequence, the NINDS-funded Anticonvulsant Screening Program (ASP; renamed Epilepsy Therapy Screening Program [ETSP] in 2015) at the University of Utah uses a stimulator designed by L.A. Woodbury (Woodbury and Davenport, 1952) to deliver currents independent of the external resistance (Swinyard, 1972; White et al., 1995). A similar stimulator was designed by the Löscher lab (Löscher et al., 1991). Anticonvulsant ED_{50}s of ASMs obtained with adequate stimulators are illustrated in Fig. S1A and Table 1.

LEV is an example of the magnitude of inter-lab differences in anticonvulsant ED_{50}s in the MES test that are related to the stimulator used. Gower et al. (1992) reported an i.p. ED_{50} of 24 mg/kg in the MES test (50 mA; corneal electrodes) in mice, while Löscher and Hönack (1993) reported that LEV is ineffective in the MES test at doses up to 500 mg/kg i.p., which was subsequently confirmed by the NINDS-funded ASP (White et al., 1995; Barton et al., 2001) and by UCB Pharma (Klitgaard et al., 1998). While Gower et al. (1992) did not specify the stimulation device used in her study, we used a stimulator which, due to its high voltage capacity (7000 V), delivers constant currents up to 200 mA regardless of the external impedance of mice or rats (Löscher and Hönack, 1993), similar to the stimulator used by the

ASP/ETSP (White et al., 1995). LEV increases the electroconvulsive threshold in mice (Löscher and Hönack, 1993); so it will be effective in the MES test at submaximal currents, explaining the huge difference in ED_{50} between Gower et al. (1992) and subsequent studies with adequate stimulators (Löscher and Hönack, 1993; White et al., 1995; Klitgaard et al., 1998). Indeed, internal testing at UCB Pharma showed that the discrepancy in LEV's efficacy in the MES test between the results of Gower et al. (1992) and other groups was related to the stimulator used in the Gower et al. study, which was unable to deliver a maximal electroshock of 50 mA to mice (Klitgaard et al., 1998).

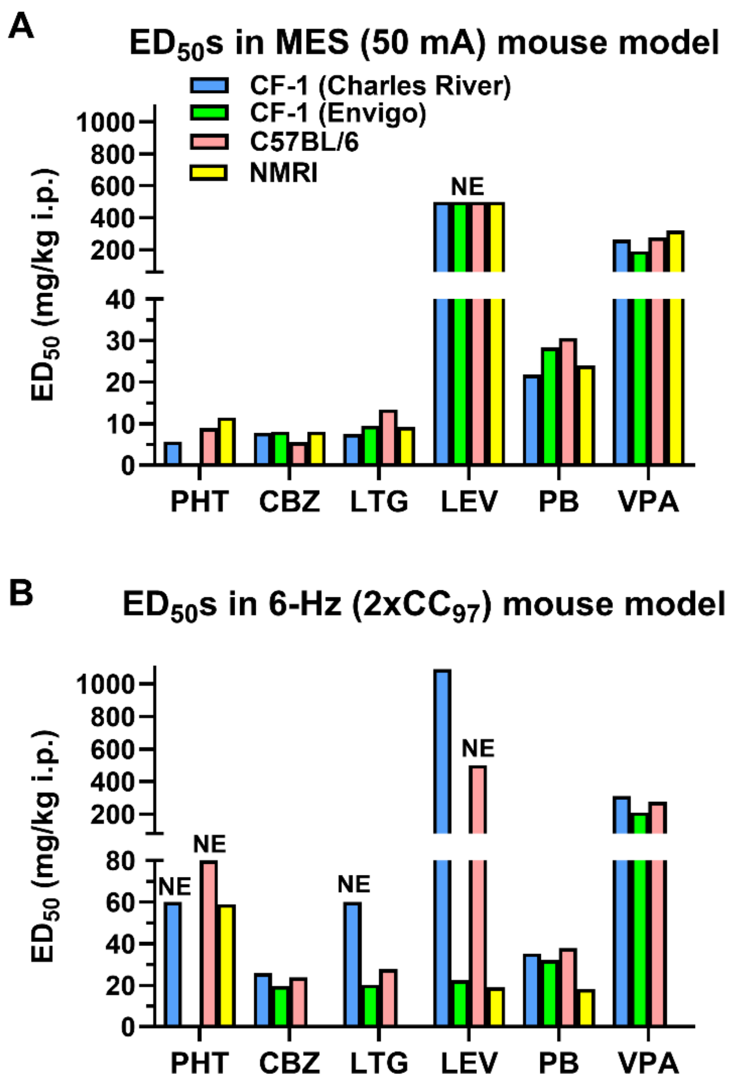

Fig. S1.

The effect of mouse strain on anticonvulsant ED_{50}s of antiseizure medications in the maximal

electroshock seizure (MES) test (A) and the 6-Hz test of focal seizures (B). A: The ED_{50} in the MES test was determined at a suprathreshold current of 50 mA. B: The ED_{50} in the 6-Hz test was determined at a suprathreshold current of twice the seizure threshold (CC_{97}), which is 44 mA in most mouse strains. Data are from Löscher et al. (1991); Barton et al. (2001); Bankstahl et al. (2013); Leclercq and Kaminski (2015); and Koneval et al. (2020). Abbreviations: CC, convulsive current; CBZ, carbamazepine; ED, effective dose; LTG, lamotrigine; LEV, levetiracetam; MES, maximal electroshock seizure; NE, not effective up to the highest dose tested; PB, phenobarbital; VPA, valproate.

Provided that an adequate stimulator is used, the sex and strain of the animals do not affect the MES test, although they may affect the electroconvulsive threshold (Löscher et al., 2017). As shown in Fig. S1A, determination of anticonvulsant ED_{50}s in the MES test is not significantly affected by the mouse strain used for performing this test, which is in sharp contrast to the 6-Hz mouse model discussed in the next section.

2. The audiogenic seizure test in mice

This test uses mice with inherited susceptibility to the induction of generalized convulsive seizures by an audiogenic stimulus, such as the audiogenic seizure-prone DBA/2 and Frings mouse strains (Löscher and Meldrum, 1984; Löscher, 1984; Faingold et al., 2017). Such seizures can also be induced in audiogenic seizure-prone rat strains such as the genetic epilepsy-prone rat (GEPR). Both DBA/2 mice and GEPR have a chronic audiogenic reflex epilepsy and, thus, are used as models for this type of epilepsy (De Sarro et al., 2017). DBA/2 mice have long been used to screen novel ASMs against generalized seizures (Chapman et al., 1984; De Sarro et al., 2017), but their use has decreased because they generally respond to almost all structurally and mechanism-wise divergent anticonvulsant drugs and do not allow for determining whether a new investigational drug possesses higher efficacy than clinically

established ASMs, particularly in difficult-to-treat types of epilepsy (Löscher, 2016). On the other hand, DBA/2 mice identified the antiseizure efficacy of LEV (Gower et al. 1992), while, as discussed above, LEV was ineffective in the MES and s.c. PTZ tests (Löscher and Hönack, 1993) and thus failed in the ASP of the NINDS in the early 1990s (Löscher et al., 2016). The DBA/2 mouse model was also decisive in identifying the antiseizure activity of brivaracetam and seletracetam (Klitgaard et al., 2016).

The complex mechanisms underlying audiogenic seizure susceptibility in DBA/2 mice have been studied extensively (Faingold et al., 2017). In addition to audiogenic seizures, these mice also display a genetically based increased susceptibility to absence seizures and handling-induced seizures. DBA/2 mice exhibit a polygenic inheritance pattern, which affects their susceptibility to audiogenic seizures (Bosco et al., 2023). In addition to using DBA/2 mice as a model of seizures, these mice are increasingly being used as a model of SUDEP (sudden unexpected death in epilepsy) (Bosco et al., 2023).

Recently, we found a highly significant correlation between the antiseizure potencies of ASMs and investigational drugs in the DBA/2 mouse model (expressed as ED_{50} in mg/kg) and the average effective oral doses of ASMs in the human photosensitivity model (Löscher and Kasteleijn-Nolst Trenité, 2025), which was the reason to include this model here. Interestingly, such a correlation was not found for the audiogenic seizure-prone rat or the photosensitive baboon. However, DBA/2 mice did not predict the failure of competitive N-methyl-D-aspartate (NMDA) receptor antagonists as novel epilepsy therapies (see section 7), but such drugs were quite potent in suppressing clonic audiogenic seizures at doses below those inducing adverse effects (Chapman et al., 1991).

3. The 6-Hz mouse model of difficult-to-treat focal seizures

In this model, the cornea is electrically stimulated for 3 sec using a low current frequency (6-Hz) that produces seizures characterized by stun, forelimb clonus, limbic seizures such as

head nodding, stereotyped chewing, and twitching of the vibrissae. The 6-Hz seizure model was developed by Toman in 1951 and designated as the "psychomotor seizure test" because the behavioral alterations resembled those seen clinically in psychomotor (i.e., complex-partial) seizures (Toman, 1951). However, subsequent studies with the relatively few available ASMs at that time indicated that the pharmacological profile of the 6-Hz test, particularly its resistance to phenytoin (PHT), was not consistent with the clinical profile of these ASMs in the treatment of psychomotor seizures (Brown et al., 1953), so the 6-Hz test was subsequently abandoned. Fifty years later, Barton et al. (2001) re-evaluated the utility of the 6-Hz model as a potential screen for drug-resistant focal epilepsy. While the test did not discriminate between clinical classes of ASMs when used at seizure threshold in CF-1 mice (CC_{97} = 22 mA), increasing the current intensity by 50% (i.e., 32 mA) decreased the sensitivity of the 6-Hz seizure to PHT and lamotrigine (LTG) (Barton et al., 2001). At a current intensity of $2 \times CC_{97}$ (i.e., 44 mA), only two ASMs, LEV and valproate (VPA), displayed complete protection against 6-Hz seizures in CF-1 mice, although the efficacy of these drugs was markedly reduced when compared to the lower stimulation intensities (Barton et al., 2001). Based on these observations, Barton et al. (2001) suggested that the 6-Hz stimulation may provide a useful model of drug-resistant focal seizures, which was the reason to include this test in the present analysis. On the other hand, the 6-Hz test does identify the anticonvulsant activity of ASMs such as LEV and tiagabine, which are ineffective in the MES test (Table 1). As a consequence, the 6-Hz model was added to the identification phase of ASP/ETSP (Smith et al., 2007). As shown in Table 1, for many ASMs, ED_{50}s in the 6-Hz (44 mA) test are considerably higher than ED_{50}s in the MES or audiogenic seizure tests, but there are exceptions, such as brivaracetam and VPA.

More recently, it has been shown that the mouse strain and substrain markedly affect the potency of ASMs in the 6-Hz model (Bankstahl et al., 2013; Leclercq and Kaminski, 2015; Koneval et al., 2020). As shown in Fig. S1B, this is particularly true for LEV and LTG.

While the ED_{50} of LEV in CF-1 mice from Charles River is >1000 mg/kg in the 6-Hz (44 mA) test (Barton et al., 2001; Metcalf et al., 2017), it is 22.5 mg/kg in CF-1 mice from Envigo, a 40-fold difference (Koneval et al., 2020). CF-1 mice are an outbred strain, so genetic differences can occur between different vendors (Löscher et al., 2017). Interestingly, a low ED_{50} (19 mg/kg) of LEV in the 6-Hz (44 mA) model was also reported for the NMRI outbred mouse strain purchased from Charles River (Leclercq and Kaminski, 2015). When inbred C57Bl/6NCrl mice (Charles River) were used, Koneval et al. (2020) reported that only 4/8 mice were protected at 500 mg/kg LEV, whereas Leclercq and Kaminski (2015) reported an ED_{50} of 254 mg/kg in C57Bl/6J mice from Charles River, which substantiates that different substrains of C57Bl/6 mice differ in their pharmacological sensitivity (Löscher et al., 2017). Leclercq and Kaminski (2015) also reported that the stimulator used for the 6-Hz model affects ED_{50}s determined in this model, indicating that not all commercial stimulators can reliably induce a current of 44 mA in mice (see also section 1). Thus, comparisons of anticonvulsant potencies in the 6-Hz mouse model across different drugs should ideally be based on the same strain/substrain of mice, using an adequate stimulator with a powerful self-adjusting stimulus voltage. In Table 1, most ED_{50}s in the 6-Hz mouse model are from the ASP/ETSP program, performed in male CF-1 mice with the same stimulator.

4. The amygdala kindling rat model of difficult-to-treat focal seizures

The MES and 6-Hz tests are performed in naïve healthy mice or rats, while amygdala kindled rats are used for drug testing after repeated kindling stimulations have induced chronic, permanent brain alterations (the fully kindled state), including increased susceptibility to electrical stimuli (Goddard et al., 1969; Sato et al., 1990). When rats are stimulated once daily by a brief (1 sec) electrical stimulus via a depth electrode in the basolateral amygdala, initially subconvulsive stimuli (e.g., 400-500 µA) instigate the induction of brief focal seizures and “afterdischarges” in the EEG recorded from the stimulation site. If daily stimulation continues

for ~2 weeks, the seizure threshold decreases and seizure severity and duration progressively increase, leading to a fully kindled state, which is marked by consistent development of generalized convulsive seizures (Sato et al., 1990). Thus, amygdala kindling is a model of focal-onset seizures, which secondarily generalize to convulsive seizures, bearing similarities to temporal lobe epilepsy (TLE)(Sato et al., 1990; McIntyre et al., 2002; Löscher and White, 2023). The different types of seizures elicited in kindled rats are rated by the Racine scale (Racine, 1972) into focal (limbic) and generalized convulsive seizures, which allows for evaluating drug efficacies against different seizure types.

Albright and Burnham (1980) were the first to report that focal seizures in the amygdala kindling model are refractory to several ASMs, including PHT, phenobarbital (PB), carbamazepine (CBZ), VPA, diazepam (DZP), and clonazepam, while generalized convulsive seizures are blocked by these drugs. They concluded that the model will be useful in screening for more effective drugs for focal seizures, which are often resistant to ASMs. These important findings of Albright and Burnham (1980) were subsequently confirmed by Löscher et al. (1986), who proposed that, based on both the behavioral and pharmacologic characteristics of the amygdala kindling model, fully kindled rats may be a useful model for drug-resistant focal seizures with secondary generalization. As shown in Fig. S2A, focal seizures in amygdala kindled rats were either resistant to treatment (primidone, DZP) or markedly less responsive to ASMs (PB, PHT, CBZ) than generalized convulsive seizures. Furthermore. Löscher et al. (1986) found that – except for DZP - the generalized convulsive seizures in kindled rats were less responsive to ASMs than such seizures in the MES test in non-kindled rats (Fig. S2A), further substantiating the idea that amygdala kindled rats are a model of drug-resistant epilepsy.

Following these initial studies, comparative analysis of preclinical and clinical findings has confirmed a high predictive validity of fully kindled animals for testing novel ASMs for the treatment of focal epilepsy (Löscher and White, 2023). Importantly, Löscher

and Hönack (1993) reported that LEV, which is ineffective in the MES model, is highly effective at blocking both focal and generalized seizures in amygdala kindled rats (Table 1), which was crucial for the decision of UCB Pharma to further develop this ASM (Löscher et al., 2016).

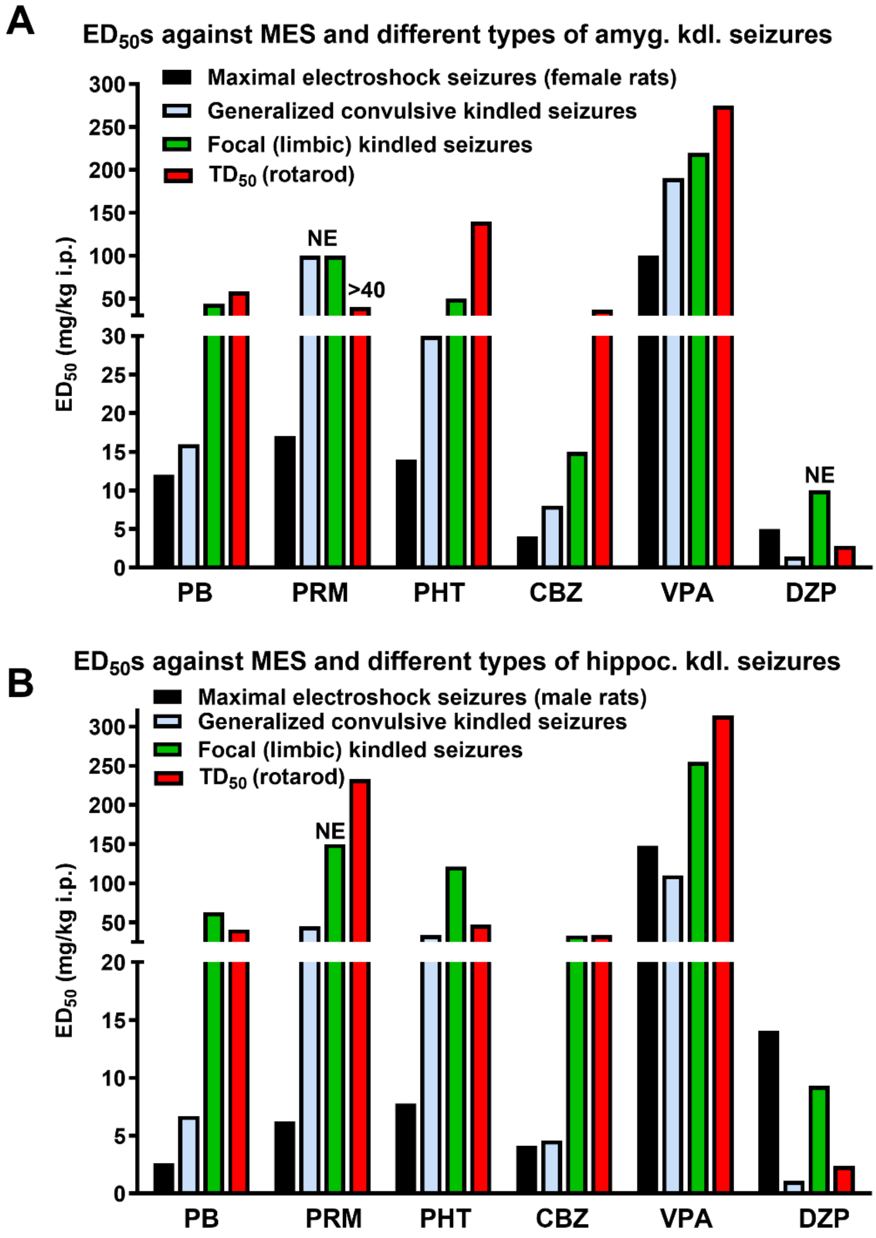

Fig. S2

Anticonvulsant ED_{50}s of antiseizure medications (ASMs) against different types of focal and generalized seizures in rats. A: ED_{50}s against (1) tonic hindlimb seizures in the maximal electroshock seizure (MES) test in normal (non-kindled) rats; (2) generalized convulsive seizures in amygdala

kindled rats; and (3) focal seizures in amygdala kindled rats. For comparison, the TD_{50} determined in the rotarod test is shown. All data are from female Wistar rats (Löscher et al., 1986; Löscher and Nolting, 1991). Note that, except for diazepam, all ASMs are markedly less effective against kindled seizures than in the MES model. B: ED_{50}s against (1) tonic hindlimb seizures in the maximal electroshock seizure (MES) test in normal (non-kindled) rats; (2) generalized convulsive seizures in hippocampal kindled rats; and (3) focal seizures in hippocampal kindled rats. For comparison, the TD_{50} determined in the rotarod test is shown. All data are from male rats (Lothman et al., 1988; Guignet et al., 2020). Note that, except for diazepam, all ASMs are markedly less effective against focal kindled seizures than against generalized seizures in the MES model. Abbreviations: CBZ, carbamazepine; DZP, diazepam; ED, effective dose; MES, maximal electroshock seizure; NE, not effective up to the highest dose tested; PB, phenobarbital; PHT, phenytoin; PRM, primidone; TD, (neuro)toxic dose; VPA, valproate.

Indeed, in contrast to other ASMs (Fig. S2A), LEV exerts about the same efficacy against focal and generalized seizures in amygdala kindled rats with ED_{50}s of 33 and 32 mg/kg, respectively (Higgins et al., 2010). Overall, the amygdala kindling model correctly predicted the clinical utility of various other ASMs against focal and secondarily generalized seizures in patients with epilepsy (Smith et al., 2007; Löscher, 2017), which was the reason to include this model in the present analysis.

Several modifications of the amygdala kindling model have been developed, including the LTG-resistant amygdala kindling model (Srivastava et al., 2013; Metcalf et al., 2019), which is used in the differentiation phase of the ETSP (Wilcox et al., 2020), and the PHT-resistant amygdala kindled rat (Löscher and Rundfeldt, 1991). For the present analysis, data from the conventional model were used.

Amygdala kindling can be affected by rat strain and sex (Löscher et al., 2017); however, the anticonvulsant ED_{50}s determined in this model are hardly affected by such factors. For instance, while the experiments of Albright and Burnham (1980) were performed

in male hooded rats, Löscher et al. (1986) used female Wistar rats. As described above, the outcome of the two studies was remarkably similar.

Amygdala kindling can also be performed in mice (McNamara et al., 1980). As recently shown for the investigational ASM padsevonil (Leclercq et al., 2020), ED_{50}s for suppression of generalized convulsive seizures in amygdala kindled rats and mice were 2.43 mg/kg (rats) and 1.2 mg/kg (mice), while the duration of focal afterdischarges was more resistant to padsevonil in mice than in rats. However, in both species, focal afterdischarges were only partially suppressed by padsevonil, predicting a low efficacy of this drug against focal seizures, which was subsequently confirmed by clinical trials (Rademacher et al., 2022).

For drug testing, fully kindled seizures are either induced by a seizure threshold current (either the afterdischarge threshold [ADT] or the generalized seizure threshold [GST]) or by a suprathreshold current (e.g., 400-500 µA). The choice of current affects the ED_{50} of ASMs that are typically more potent at seizure threshold than suprathreshold current, which is similar to the MES and 6-Hz models (see above). Thus, an adequate comparison of different ASMs is only possible if the same (preferably suprathreshold) current has been used in all experiments. Furthermore, in addition to drug testing at suprathreshold current, often a drug's effect on GST and ADT is determined as a measure of a drug's efficacy on focal vs. generalized seizures.

Unfortunately, only relatively few laboratories determine anticonvulsant ED_{50}s in the amygdala kindling model separately against focal vs. secondary generalized convulsive seizures (Table 1). Even worse, some groups only determine ED_{50}s against the generalized convulsive seizures, thus missing the opportunity to predict drug efficacies against difficult-to-treat focal seizures. Again, padsevonil is an example because ED_{50}s in amygdala kindled rats and mice were only determined for the generalized convulsive seizures (Leclercq et al., 2020), thus obviously leading to false positive data.

5. The hippocampal kindling rat model of difficult-to-treat focal seizures

The kindling phenomenon is not restricted to electrical stimulation of the amygdala but also occurs following stimulation of other limbic or neocortical sites (McNamara et al., 1980). Lothman et al. (1985) described a protocol of "rapid kindling" in which 10-sec trains of suprathreshold tetanic electrical stimuli are delivered every few minutes to the hippocampus, resulting in a stable, fully kindled state within 2 days, thus markedly shortening the time needed to reach the fully kindled state with conventional amygdala kindling. In a subsequent study, Lothman et al. (1988) used this model of rapidly recurring hippocampal seizures (RRHS) for testing of ASMs. Whereas in amygdala kindled rats, each rat is usually only used for 1-2 acute drug treatments per week, the RRHS model allows for performing dose response experiments in a single study period of one day (Lothman et al., 1988). As shown in Fig. S2B, the outcome of drug studies in the RRHS model was similar to the respective data in amygdala kindled rats (Fig. S2A) in that focal (limbic) seizures were markedly less responsive than generalized convulsive seizures. For comparison, we also show MES ED_{50}s and median toxic doses (TD_{50}s) in nonkindled male rats in Fig. S2B, indicating that, except for CBZ and DZP, ED_{50}s in the MES test are lower than ED_{50}s against generalized convulsive seizures in hippocampal kindled rats.

The RRHS model was used for a few years in the ETSP program but was recently replaced by the LTG-resistant amygdala kindled rat (Wilcox et al., 2020). The RRHS model was included in the present analysis because it was used by the ETSP to characterize the preclinical profile of cenobamate (CNB)(Bialer et al., 2009; Melnick et al., 2023), a novel ASM with remarkably high efficacy in patients with drug-resistant focal seizures (Klein et al., 2024).

Racine et al. (1973) examined the effect of changing the interstimulus interval on amygdala kindling. They found that the stimuli could be applied as rapidly as once an hour

without changing the number of stimuli needed for kindling. However, Löscher and Hönack (1990) reported that the interstimulation interval affects the potency of ASMs in this model. When the effects of CBZ (15 mg/kg) were determined with 4 different stimulation regimes, it was found that the anticonvulsant potency of the drug was higher in experiments with short interstimulation intervals compared to conventional protocols with interstimulation intervals of 1-3 days, indicating synergistic effects between the drug and postictal inhibition. Indication for such synergism was also found when the animals were only stimulated once daily during the drug experiments. With higher doses of CBZ or PB, the difference between the stimulation protocols was less marked. It is likely that the short interstimulation intervals used in the RRHS model also lead to overestimation of anticonvulsant potency. For instance, as shown in Fig. S2A, both focal and generalized amygdala kindled seizures were resistant to primidone, whereas generalized seizures of the RRHS model responded to this drug (Fig. S2B). Similarly, focal amygdala kindled seizures were resistant to DZP, whereas focal seizures of the RRHS model responded to this drug. The possible interaction between anticonvulsant effects and postictal inhibitory processes developing during multistimulation protocols, which was not assessed by Lothman et al. (1988), should be kept in mind when using the RRHS model.

Similar to the amygdala kindling model, drugs are tested in the hippocampal kindling model either at a suprathreshold current or at ADT or GST. This is important when comparing the antiseizure efficacy of different drugs in this model because seizures induced by threshold currents are more easily suppressed than seizures elicited at suprathreshold currents.

6. The pilocarpine rat model of difficult-to-treat focal seizures

The acute seizures induced by the cholinergic muscarinic agonist pilocarpine (PILO) in rodents have been proposed, on the basis of EEG monitoring, behavioral analysis, and morphological sequelae, as an animal model resembling some aspects of human TLE (Turski

et al., 1983; Turski et al., 1984; Holtzman and Lowenstein, 1995; Leite et al., 2002). Depending on the dose of PILO administered in rats or mice, animals develop focal (limbic) seizures (resembling focal kindled seizures) that generalize to convulsive seizures and status epilepticus (SE). At high doses that induce SE, the animals develop spontaneous recurrent seizures (SRS) in the weeks after SE. Thus, the PILO model can be used for drug testing in three different ways: (1) treatment with investigational drugs before PILO to suppress the acute seizures and SE; (2) treatment after SE onset to terminate the SE; and (3) prolonged treatment during the chronic phase to suppress the SRS. For the present analysis, only the first option will be examined.

A modification of the PILO model consists of pretreatment with lithium (Li), which markedly reduces the dose of PILO needed to induce seizures and SE (Honchar et al., 1983; Curia et al., 2008), most likely by depletion of brain inositol (Belmaker and Bersudsky, 2007) and peripheral inflammatory changes that alter the permeability of the blood-brain barrier to PILO (Vezzani, 2009). In terms of EEG, behavioral, and morphological sequelae, the PILO and Li-PILO models are very similar but markedly differ in the responsiveness to ASMs (see below). Unfortunately, the mortality of the PILO and Li-PILO models is high but can be reduced by repeated i.p. administration of 10 mg/kg PILO at 30-min intervals instead of one high bolus injection (Glien et al., 2001).

Turski et al. (1987) were the first to describe the effect of ASMs on the seizures induced by PILO. Clonazepam suppressed limbic and motor seizures induced by 380 mg/kg PILO with an ED_{50} of 0.35 mg/kg. Similarly, PB and VPA protected the rats from seizures with ED_{50}s of 23.4 mg/kg and 286 mg/kg, respectively. In contrast, PHT (10-200 mg/kg) and CBZ (10-50 mg/kg) did not suppress seizures produced by PILO. In addition to the anticonvulsant effect, clonazepam, PB, and VPA, but not PHT and CBZ, prevented or reduced the brain damage induced by PILO. These results indicate that only certain ASMs prevent PILO-induced seizures and prevent the occurrence of seizure-related brain damage.

Turski et al. (1987) concluded that the resistance of seizures produced by PILO in rats to ASMs reaffirms the clinically obvious lack of effective treatments for focal (limbic) seizures.

In a subsequent study by Sofia et al. (1993), the effects of ASMs were determined in the PILO and Li-PILO models in rats. As shown in Fig. S3, when seizures and SE were induced by either PILO (380 mg/kg) or PILO (30 mg/kg) after pretreatment with lithium (3 mEq/kg), striking differences in anticonvulsant ED_{50}s were found for CBZ, felbamate (FBM), and diazepam, but not PB, PHT, and VPA. PHT was ineffective in both models, while CBZ was ineffective only in the PILO model (consistent with the findings of Turski et al. [1987]) but prevented seizures and SE in the Li-PILO model with an ED_{50} of 28.9 mg/kg. PB was effective in both models with ED_{50}s of 16.2 (PILO) and 15.1 (Li-PILO) mg/kg, respectively. Similarly, VPA exhibited similar efficacy in both models (Fig. S3). In contrast, DZP was 6-times more potent in the PILO model (ED_{50} 0.75 mg/kg) than in the Li-PILO model (4.5 mg/kg). The largest potency difference was determined for FBM with ED_{50}s of 799 mg/kg in the PILO and 80.5 mg/kg in the Li-PILO model.

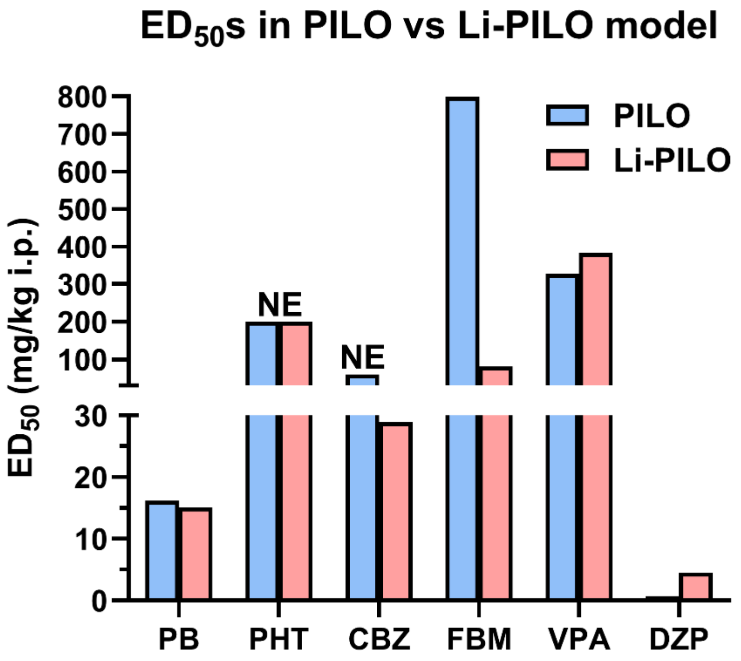

Fig. S3

Anticonvulsant ED_{50}s of antiseizure medications in the pilocarpine vs. lithium-pilocarpine rat models. Drugs were administered 15-60 min prior to pilocarpine injection in male Sprague-Dawley rats. Administration of pilocarpine (380 mg/kg i.p.) or pilocarpine (30 mg/kg s.c.) 19 h after lithium (3 mEq/kg) produced limbic (focal) and generalized convulsive seizures and status epilepticus in 90% of the rats. Data are from Sofia et al. (1993). Abbreviations: CBZ, carbamazepine; DZP, diazepam; ED, effective dose; FBM, felbamate; NE, not effective up to the highest dose tested; PB, phenobarbital; PHT, phenytoin; VPA, valproate.

The authors suggested that – despite the similarity in behavioral seizures produced by both treatments – different biochemical mechanisms may be involved. For instance, it was suggested that activation of NMDA receptors is an important factor in SE and brain damage in the Li-PILO model. This suggestion is based on experiments with the NMDA receptor antagonist MK-801 (dizocilpine), which prevented the development of SE in the Li-PILO model but not in rats treated with PILO alone (Ormandy et al., 1989). At least in part, FBM is thought to act via antagonism of NMDA receptors (Rogawski et al., 2016), which may explain the 10-fold difference in ED_{50} between the PILO and Li-PILO models determined by Sofia et al. (1993). However, NMDA receptors are thought to be involved in the PILO-only model as well (Curia et al., 2009).

Like other animal models, the PILO and Li-PILO models are affected by rodent strain/substrain and sex, although strain differences are more marked in mice than in rats (Curia et al., 2008; Löscher et al., 2017). For instance, Turski et al. (1987) used Wistar rats, while Sprague-Dawley rats were used by Sofia et al. (1993), but the outcome of the pharmacological studies was quite similar.

The Li-PILO model was used in the ETSP for a few years to test drugs for their potential as countermeasures against a chemical attack. The model was included in the present analysis because it differentiated CNB from CRS (Table 1), which was pivotal for the

decision to develop CNB (Bialer et al., 2009) and correctly predicted the different efficacy of the two ASMs in add-on clinical trials in patients with drug-resistant focal seizures (Löscher et al., 2021).

7. The rotarod test in mice and rats

Drug-induced neurological deficit, such as impaired motor function, can be detected and quantified by standardized tests, such as the rotarod test in mice and rats, in which the animals are placed on a rotating plastic rod (Dunham and Miya, 1957). Quantification of motor impairment (i.e., the number of animals failing the rotarod test within a single treatment group) across several doses for a test compound allows for the determination of a median neurotoxic dose (TD_{50}), and when compared to the anticonvulsant ED_{50}, allows for calculation of the protective index (PI; therapeutic index, TD_{50}/ED_{50}). The rotarod test provides a rough assessment of tolerability, with emphasis on the potential for candidate drugs to affect motor function. New investigational ASMs, which in mice and rats exert anticonvulsant effects only at 'neurotoxic' doses ($ED_{50} \geq TD_{50}$), are considered non-selective and should not be advanced to further evaluation (Löscher and Schmidt, 1988). A PI of 2 or more is generally considered to indicate a sufficient safety ratio (Krall et al., 1978b; Wilcox et al., 2020). However, several clinically established ASMs, e.g., VPA or perampanel (Table 1), do have PIs below 2, so ranking of ASMs by their PIs, as often done in the literature, is not particularly useful. Furthermore, chronic brain alterations, such as those occurring during epileptogenesis, may alter the tolerability of drugs, which was first reported for drugs that block the N-methyl-D-aspartate (NMDA) receptor subtype of glutamate receptors (Löscher and Hönack, 1991). Such drugs were well tolerated in nonepileptic rats and healthy volunteers, but exerted severe psychotic adverse effects in amygdala kindled rats and patients with focal DRE (Löscher and Schmidt, 1994). Subsequently, kindled rats were shown to be more sensitive than non-kindled rats to CNS adverse effects of several other ASMs and

investigational compounds (Hönack and Löscher, 1995; Klitgaard et al., 2002). Thus, kindled or epileptic rodents should be included in the assessment of tolerability. Furthermore, in addition to the rotarod test, functional observation batteries such as the Irwin test should be included in preclinical evaluation of adverse effects (Fonck et al., 2015; Wilcox et al., 2020). However, for the present analysis, only the "minimal neurotoxicity" as determined by the rotarod test in non-kindled rodents and expressed by TD_{50} values is shown in Fig. S2 and Table 1.

In summary, the rotarod test provides a rough assessment of tolerability, with emphasis on the potential for candidate drugs to affect motor function. However, tolerability assessment focused primarily on motor function may limit the extrapolation of rodent data to humans (Wilcox et al., 2020).

References

Albright PS, Burnham WM. Development of a new pharmacological seizure model: effects of anticonvulsants on cortical- and amygdala-kindled seizures in the rat. *Epilepsia* 1980;21:681-9.

Bankstahl M, Bankstahl JP, Löscher W. Pilocarpine-induced epilepsy in mice alters seizure thresholds and the efficacy of antiepileptic drugs in the 6-Hertz psychomotor seizure model. *Epilepsy Res* 2013;107:205-16.

Barton ME, Klein BD, Wolf HH, White HS. Pharmacological characterization of the 6 Hz psychomotor seizure model of partial epilepsy. *Epilepsy Res* 2001;47:217-28.

Belmaker RH, Bersudsky Y. Lithium-pilocarpine seizures as a model for lithium action in mania. *Neurosci Biobehav Rev* 2007;31:843-9.

Bialer M, Johannessen SI, Levy RH, Perucca E, Tomson T, White HS. Progress report on new antiepileptic drugs: a summary of the Ninth Eilat Conference (EILAT IX). *Epilepsy Res* 2009;83:1-43.

Bialer M, White HS. Key factors in the discovery and development of new antiepileptic drugs. *Nat Rev Drug Discov* 2010;9:68-82.

Bosco F, Guarnieri L, Leo A, Tallarico M, Gallelli L, Rania V *et al.* Audiogenic epileptic DBA/2 mice strain as a model of genetic reflex seizures and SUDEP. *Front Neurol* 2023;14:1223074.

Brown WC, Schiffman DO, Swinyard EA, Goodman LS. Comparative assay of antiepileptic drugs by "pychomotor" seizure test and minimal electroshock threshold test. *J Pharmacol Exp Ther* 1953;107:273-83.

Castel-Branco MM, Alves GL, Figueiredo IV, Falcao AC, Caramona MM. The maximal electroshock seizure (MES) model in the preclinical assessment of potential new antiepileptic drugs. *Methods Find Exp Clin Pharmacol* 2009;31:101-6.

Chapman AG, Croucher MJ, Meldrum BS. Evaluation of anticonvulsant drugs in DBA/2 mice with sound-induced seizures. *Arzneim -Forsch (Drug Res)* 1984;34:1261-70.

Chapman AG, Graham JL, Patel S, Meldrum BS. Anticonvulsant activity of two orally active competitive N-methyl-D-aspartate antagonists, CGP 37849 and CGP 39551, against sound-induced seizures in DBA/2 mice and photically induced myoclonus in *Papio papio*. *Epilepsia* 1991;32:578-87.

Curia G, Longo D, Biagini G, Jones RS, Avoli M. The pilocarpine model of temporal lobe epilepsy. *J Neurosci Methods* 2008;172:143-57.

De Sarro G, Russo E, Citraro R, Meldrum BS. Genetically epilepsy-prone rats (GEPRs) and DBA/2 mice: Two animal models of audiogenic reflex epilepsy for the evaluation of new generation AEDs. *Epilepsy Behav* 2017;71:165-73.

Dunham NW, Miya TS. A note on a simple apparatus for detecting neurological deficit in rats and mice. *J Am Pharm Assoc* 1957;46:208-9.

Faingold C, Tupal S, N'Gouemo P. Genetic Models of Reflex Epilepsy and SUDEP in Rats and Mice. In: Pitkänen A, Buckmaster PS, Galanopoulou AS, Moshé SL, eds. *Models of seizures and epilepsy. 2nd edition.* London: Academic Press, 2017:441-53.

Fonck C, Easter A, Pietras MR, Bialecki RA. CNS Adverse Effects: From Functional Observation Battery/Irwin Tests to Electrophysiology. *Handb Exp Pharmacol* 2015;229:83-113.

Glien M, Brandt C, Potschka H, Voigt H, Ebert U, Löscher W. Repeated low-dose treatment of rats with pilocarpine: low mortality but high proportion of rats developing epilepsy. *Epilepsy Res* 2001;46:111-9.

Goddard GV, McIntyre DC, Leech CK. A permanent change in brain function resulting from daily electrical stimulation. *Exp Neurol* 1969;25:295-330.

Gower AJ, Noyer M, Verloes R, Gobert J, Wülfert E. ucb L059, a novel anti-convulsant drug: pharmacological profile in animals. *Eur J Pharmacol* 1992;222:193-203.

Guignet M, Campbell A, White HS. Cenobamate (XCOPRI®): Can preclinical and clinical evidence provide insight into its mechanism of action? *Epilepsia* 2020;61:2329-39.

Higgins GA, Breysse N, Undzys E, Derksen DR, Jeffrey M, Scott BW *et al.* Comparative study of five antiepileptic drugs on a translational cognitive measure in the rat: relationship to antiepileptic property. *Psychopharmacology (Berl)* 2010;207:513-27.

Holtzman DM, Lowenstein DH. Selective inhibition of axon outgrowth by antibodies to NGF in a model of temporal lobe epilepsy. *J Neurosci* 1995;15:7062-70.

Honchar MP, Olney JW, Sherman WR. Systemic cholinergic agents induce seizures and brain damage in lithium-treated rats. *Science* 1983;220:323-5.

Hönack D, Löscher W. Kindling increases the sensitivity of rats to adverse effects of certain antiepileptic drugs. *Epilepsia* 1995;36:763-71.

Klitgaard H, Matagne A, Gobert J, Wulfert E. Evidence for a unique profile of levetiracetam in rodent models of seizures and epilepsy. *Eur J Pharmacol* 1998;353:191-206.

Klitgaard H, Matagne A, Lamberty Y. Use of epileptic animals for adverse effect testing.

Epilepsy Res 2002;50:55-65.

Klitgaard H, Matagne A, Nicolas JM, Gillard M, Lamberty Y, De Ryck M *et al.* Brivaracetam: Rationale for discovery and preclinical profile of a selective SV2A ligand for epilepsy treatment. *Epilepsia* 2016;57:538-48.

Koneval Z, Knox KM, Memon A, Zierath DK, White HS, Barker-Haliski M. Antiseizure drug efficacy and tolerability in established and novel drug discovery seizure models in outbred vs inbred mice. *Epilepsia* 2020;61:2022-34.

Krall RL, Penry JK, Kupferberg HJ, Swinyard EA. Antiepileptic drug development: I. History and a program for progress. *Epilepsia* 1978;19:393-408.

Krall RL, Penry JK, White BG, Kupferberg HJ, Swinyard EA. Antiepileptic drug development: II. Anticonvulsant drug screening. *Epilepsia* 1978;19:409-28.

Leclercq K, Kaminski RM. Genetic background of mice strongly influences treatment resistance in the 6 Hz seizure model. *Epilepsia* 2015;56:310-8.

Leclercq K, Matagne A, Provins L, Klitgaard H, Kaminski RM. Pharmacological profile of the antiepileptic drug candidate padsevonil - characterization in rodent seizure and epilepsy models. *J Pharmacol Exp Ther* 2020;372:11-20.

Leite JP, Garcia-Cairasco N, Cavalheiro EA. New insights from the use of pilocarpine and kainate models. *Epilepsy Res* 2002;50:93-103.

Lothman EW, Hatlelid JM, Zorumski CF, Conry JA, Moon PF, Perlin JB. Kindling with rapidly recurring hippocampal seizures. *Brain Res* 1985;360:83-91.

Lothman EW, Salerno RA, Perlin JB, Kaiser DL. Screening and characterization of antiepileptic drugs with rapidly recurring hippocampal seizures in rats. *Epilepsy Res* 1988;2:367-79.

Löscher W, Meldrum BS. Evaluation of anticonvulsant drugs in genetic animal models of epilepsy. *Fed Proc* 1984;43:276-84.

Löscher W. Genetic animal models of epilepsy as a unique resource for the evaluation of anticonvulsant drugs. A review. *Methods Findings Experiment Clin Pharmacol* 1984;6:531-47.

Löscher W, Jäckel R, Czuczwar SJ. Is amygdala kindling in rats a model for drug-resistant partial epilepsy? *Exp Neurol* 1986;93:211-26.

Löscher W, Schmidt D. Which animal models should be used in the search for new antiepileptic drugs? A proposal based on experimental and clinical considerations. *Epilepsy Res* 1988;2:145-81.

Löscher W, Hönack D. The effect of interstimulation interval on the assessment of anticonvulsant drug potency in fully kindled rats. *Epilepsy Res* 1990;7:182-96.

Löscher W, Hönack D. Responses to NMDA receptor antagonists altered by epileptogenesis. *Trends Pharmacol Sci* 1991;12:52.

Löscher W, Nolting B. The role of technical, biological and pharmacological factors in the laboratory evaluation of anticonvulsant drugs. IV. Protective indices. *Epilepsy Res* 1991;9:1-10.

Löscher W, Fassbender CP, Nolting B. The role of technical, biological and pharmacological factors in the laboratory evaluation of anticonvulsant drugs. II. Maximal electroshock seizure models. *Epilepsy Res* 1991;8:79-94.

Löscher W, Rundfeldt C. Kindling as a model of drug-resistant partial epilepsy: selection of phenytoin-resistant and nonresistant rats. *J Pharmacol Exp Ther* 1991;258:483-9.

Löscher W, Hönack D. Profile of ucb L059, a novel anticonvulsant drug, in models of partial and generalized epilepsy in mice and rats. *Eur J Pharmacol* 1993;232:147-58.

Löscher W, Schmidt D. Strategies in antiepileptic drug development: is rational drug design superior to random screening and structural variation? *Epilepsy Res* 1994;17:95-134.

Löscher W. Critical review of current animal models of seizures and epilepsy used in the discovery and development of new antiepileptic drugs. *Seizure* 2011;20:359-68.

Löscher W. Fit for purpose application of currently existing animal models in the discovery of novel epilepsy therapies. *Epilepsy Res* 2016;126:157-84.

Löscher W, Gillard M, Sands ZA, Kaminski RM, Klitgaard H. Synaptic Vesicle Glycoprotein 2A Ligands in the Treatment of Epilepsy and Beyond. *CNS Drugs* 2016;30:1055-77.

Löscher W, Ferland RJ, Ferraro TN. The relevance of inter- and intrastrain differences in mice and rats and their implications for models of seizures and epilepsy. *Epilepsy Behav* 2017;73:214-35.

Löscher W. Animal Models of Seizures and Epilepsy: Past, Present, and Future Role for the Discovery of Antiseizure Drugs. *Neurochem Res* 2017;42:1873-88.

Löscher W, Sills GJ, White HS. The ups and downs of alkyl-carbamates in epilepsy therapy: How does cenobamate differ? *Epilepsia* 2021;62:596-614.

Löscher W, White HS. Animal models of drug-resistant epilepsy as tools for deciphering the cellular and molecular mechanisms of pharmacoresistance and discovering more effective treatments. *Cells* 2023;12:1233.

Löscher W, Kasteleijn-Nolst Trenité D. The human photosensitive epilepsy model for clinical proof-of-principle trials of novel antiseizure medications. 2. Analysis of drug trials and predictive values of the model. *Epilepsia* 2025;66:2619-38.

McIntyre DC, Poulter MO, Gilby K. Kindling: some old and some new. *Epilepsy Res* 2002;50:79-92.

McNamara JO, Byrne MC, Dasheiff RM, Fitz JG. The kindling model of epilepsy: a review. *Prog Neurobiol* 1980;15:139-59.

Melnick SM, Shin Y, Glenn KJ. Anticonvulsant effects of cenobamate in chemically and electrically induced seizure models in rodents. *Heliyon* 2023;9:e18920.

Metcalf CS, West PJ, Thomson KE, Edwards SF, Smith MD, White HS *et al.* Development and pharmacologic characterization of the rat 6 Hz model of partial seizures. *Epilepsia* 2017;58:1073-84.

Metcalf CS, Huff J, Thomson KE, Johnson K, Edwards SF, Wilcox KS. Evaluation of antiseizure drug efficacy and tolerability in the rat lamotrigine-resistant amygdala kindling model. *Epilepsia Open* 2019;4:452-63.

Ormandy GC, Jope RS, Snead OC, III. Anticonvulsant actions of MK-801 on the lithium-pilocarpine model of status epilepticus in rats. *Exp Neurol* 1989;106:172-80.

Racine RJ. Modification of seizure activity by electrical stimulation: II. Motor seizure. *Electroenceph Clin Neurophysiol* 1972;32:281-94.

Rademacher M, Toledo M, Van Paesschen W, Liow KK, Milanov IG, Esch ML *et al.* Efficacy and safety of adjunctive padsevonil in adults with drug-resistant focal epilepsy:

Results from two double-blind, randomized, placebo-controlled trials. *Epilepsia Open* 2022;7:758-70.

Rogawski MA, Löscher W, Rho JM. Mechanisms of Action of Antiseizure Drugs and the Ketogenic Diet. *Cold Spring Harb Perspect Med* 2016;6: pii: a022780.

Sato M, Racine RJ, McIntyre DC. Kindling: basic mechanisms and clinical validity. *Electroenceph Clin Neurophysiol* 1990;76:459-72.

Smith M, Wilcox KS, White HS. Discovery of antiepileptic drugs. *Neurotherapeutics* 2007;4:12-7.

Sofia RD, Gordon R, Gels M, Diamantis W. Effects of felbamate and other anticonvulsant drugs in two models of status epilepticus in the rat. *Res Commun Chem Pathol Pharmacol* 1993;79:335-41.

Srivastava AK, Alex AB, Wilcox KS, White HS. Rapid loss of efficacy to the antiseizure drugs lamotrigine and carbamazepine: a novel experimental model of pharmacoresistant epilepsy. *Epilepsia* 2013;54:1186-94.

Swinyard EA. Electrically induced convulsions. In: Purpura DP, Penry JK, Tower D, Woodbury DM, Walter R, eds. *Experimental models of epilepsy - A manual for the laboratory worker.* New York: Raven Press, 1972:433-58.

Toman JEP, Swinyard EA, Goodman LS. Properties of maximal seizures and their alteration by anticonvulsant drugs and other agents. *J Neurophysiol* 1946;9:231-9.

Toman JEP. Neuropharmacologic Considerations in Psychic Seizures. *Neurology* 1951;1:444-60.

Turski WA, Cavalheiro EA, Schwarz M, Czuczwar SJ, Kleinrok Z, Turski L. Limbic seizures produced by pilocarpine in rats: behavioural, electroencephalographic and neuropathological study. *Behav Brain Res* 1983;9:315-35.

Turski WA, Cavalheiro EA, Bortolotto ZA, Mello LM, Schwarz M, Turski L. Seizures produced by pilocarpine in mice: a behavioral, electroencephalographic and morphological analysis. *Brain Res* 1984;321:237-53.

Turski WA, Cavalheiro EA, Coimbra C, da Penha BM, Ikonomidou-Turski C, Turski L. Only certain antiepileptic drugs prevent seizures induced by pilocarpine. *Brain Res* 1987;434:281-305.

Vezzani A. Pilocarpine-induced seizures revisited: what does the model mimic? *Epilepsy Curr* 2009;9:146-8.

White HS, Woodhead JH, Franklin MR, Swinyard EA, Wolf HH. Experimental selection, quantification, and evaluation of antiepileptic drugs. In: Levy RH, Mattson RH, Meldrum BS, eds. *Antiepileptic drugs. Fourth edition.* New York: Raven Press, 1995:99-110.

Wilcox KS, West PJ, Metcalf CS. The Current Approach of the Epilepsy Therapy Screening Program Contract Site for Identifying Improved Therapies for the Treatment of Pharmacoresistant Seizures in Epilepsy. *Neuropharmacology* 2020;166:107811.
