# Supplementary material for: Ranking of antiseizure medications in a panel of focal seizure models predicts their comparative efficacy in clinical add‐on trials in drug‐resistant focal epilepsy
Source: Epilepsia. 2026 Mar 28;67(7):3719–37. doi: 10.1002/epi.70210 (PMC13360943; doi:10.1002/epi.70210)
Supplement: Supplementary file 2 — Appendix S2. [file EPI-67-3719-s003.pdf]

Appendix S2

Evaluation of drug potency vs. efficacy in animal models

The concepts of drug potency and efficacy are often confused and used interchangeably within the scientific community and the pharmaceutical industry (Waldman, 2002). Potency and efficacy are two distinct pharmacological concepts used to compare medications such as ASMs. Potency refers to the dose of a drug required to produce a defined effect (e.g., seizure suppression in 50% of the animals), while efficacy refers to the maximum effect (E_{max}) that a drug can produce, regardless of dose. Thus, as shown in Fig. S4, a drug may have a lower potency but higher efficacy than another drug in the same model. In epilepsy treatment, drugs with higher efficacy are preferred for controlling seizures, but their potency will determine the starting dose and adjustments needed (Ventola, 2014).

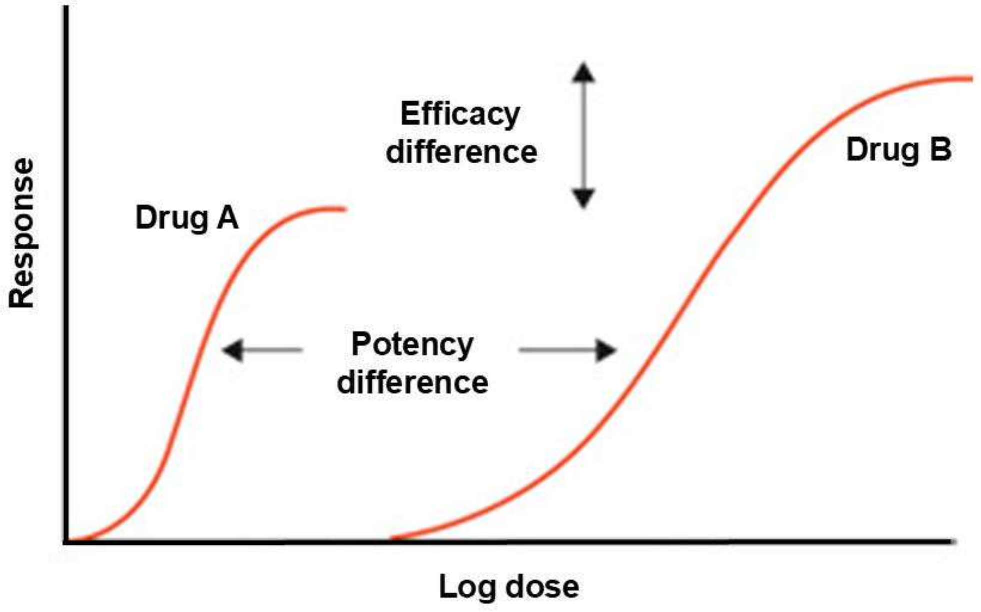

Fig. S4

The concept of drug potency vs. efficacy. Although drug B has a lower potency than drug A concerning the dose required to produce a defined effect (e.g., a 50% response), the maximal efficacy of drug B is higher, i.e., drug B produces a larger maximal response than drug A.

Anticonvulsant ED_{50}s of drugs in animal seizure models, as used for the present analysis, are thought to primarily relate to drug potency and not efficacy (Löscher, 2011). Anti-seizure ED_{50}s, i.e., the dose suppressing seizures in 50% of the animals, are calculated from dose-response curves, testing one group of animals per dose. The lower the ED_{50}, the more potent the drug, and high potency is often an important argument for selecting drugs for further development. However, it is the antiseizure efficacy that finally determines the clinical usefulness of a new ASM and should be considered during preclinical drug testing (Löscher and White, 2023).

While preclinical drug potency can be a good preclinical marker of the therapeutic potential of a drug (Waldman, 2002), it does not necessarily predict clinical efficacy. One recent example is the clinical efficacy of padsevonil and cenobamate (CNB), which are both highly potent in diverse animal models of seizures and epilepsy (Leclercq et al., 2020; Melnick et al., 2023); however, while add-on treatment with CNB proved to be highly effective in several clinical trials in patients with drug-resistant focal epilepsy (Klein et al., 2024), padsevonil failed in such trials (Rademacher et al., 2022).

In animal models of drug-resistant focal seizures, efficacy is more difficult to determine than potency, but one approach is determining ED_{50}s in the 6-Hz model at increasing current intensities (CC_{97}, 1.5xCC_{97}, 2xCC_{97}) as proposed by Barton et al. (2001). At threshold current (CC_{97}), most ASMs block seizures in this model. However, when the threshold current is increased, most ASMs become less effective or ineffective in this test. Only a few second- and third-generation ASMs, in particular CNB, remain effective (Guignet et al., 2020), thus allowing the differentiation of ASMs in terms of anti-seizure efficacy in this mouse model.

An alternative view of anticonvulsant ED_{50}s being a pure measure of potency is the fact that often an increase in seizure threshold is involved in a drug's ED_{50} in a seizure model. For instance, LEV significantly increases the electroconvulsive threshold for tonic seizures in

mice and rats, but the increase is not sufficiently large to allow the drug to be effective in the MES test with suprathreshold stimulation (Löscher and Hönack, 1993). Thus, the resistance of the MES test to LEV (See Appendix S1) results from the low efficacy of LEV to increase seizure threshold in this model. In contrast, LEV induces large increases in the ADT and GST in amygdala kindled rats (Löscher and Hönack, 1993; Löscher et al., 2016), so that seizures induced by suprathreshold stimulation in this model are blocked by LEV (Table 1). Thus, the low ED_{50} of LEV in amygdala kindled rats results from a high efficacy to increase seizure threshold. Many ASMs act by increasing seizure threshold (Löscher and Schmidt, 1988; Löscher, 2016). As a consequence, the ED_{50}s in the diverse models shown in Table 1 may reflect both potency and efficacy, and resistance of a model to an ASM primarily relates to insufficient efficacy in this model. The possibility that the ED_{50} may be a surrogate of efficacy seems to be substantiated by the significant correlations with clinical efficacy reported here. However, it is currently not clear whether this also applies to ASMs that are more potent than CNB and LEV in animal models but yet did not approach the clinical efficacy of CNB, as measured by seizure freedom.

References

Barton ME, Peters SC, Shannon HE. Comparison of the effect of glutamate receptor modulators in the 6 Hz and maximal electroshock seizure models. *Epilepsy Res* 2003;56:17-26.

Guignet M, Campbell A, White HS. Cenobamate (XCOPRI®): Can preclinical and clinical evidence provide insight into its mechanism of action? *Epilepsia* 2020;61:2329-39.

Klein P, Friedman D, Kwan P. Recent Advances in Pharmacologic Treatments of Drug-Resistant Epilepsy: Breakthrough in Sight. *CNS Drugs* 2024;38:949-60.

Leclercq K, Matagne A, Provins L, Klitgaard H, Kaminski RM. Pharmacological profile of the antiepileptic drug candidate padsevonil - characterization in rodent seizure and epilepsy models. *J Pharmacol Exp Ther* 2020;372:11-20.

Löscher W, Schmidt D. Which animal models should be used in the search for new antiepileptic drugs? A proposal based on experimental and clinical considerations. *Epilepsy Res* 1988;2:145-81.

Löscher W, Hönack D. Profile of ucb L059, a novel anticonvulsant drug, in models of partial and generalized epilepsy in mice and rats. *Eur J Pharmacol* 1993;232:147-58.

Löscher W. Critical review of current animal models of seizures and epilepsy used in the discovery and development of new antiepileptic drugs. *Seizure* 2011;20:359-68.

Löscher W, Gillard M, Sands ZA, Kaminski RM, Klitgaard H. Synaptic Vesicle Glycoprotein 2A Ligands in the Treatment of Epilepsy and Beyond. *CNS Drugs* 2016;30:1055-77.

Löscher W. Fit for purpose application of currently existing animal models in the discovery of novel epilepsy therapies. *Epilepsy Res* 2016;126:157-84.

Löscher W, White HS. Animal Models of Drug-Resistant Epilepsy as Tools for Deciphering the Cellular and Molecular Mechanisms of Pharmacoresistance and Discovering More Effective Treatments. *Cells* 2023;12:1233.

Melnick SM, Shin Y, Glenn KJ. Anticonvulsant effects of cenobamate in chemically and electrically induced seizure models in rodents. *Heliyon* 2023;9:e18920.

Rademacher M, Toledo M, Van Paesschen W, Liow KK, Milanov IG, Esch ML *et al.* Efficacy and safety of adjunctive padsevonil in adults with drug-resistant focal epilepsy: Results from two double-blind, randomized, placebo-controlled trials. *Epilepsia Open* 2022;7:758-70.

Ventola CL. Epilepsy management: newer agents, unmet needs, and future treatment strategies. *P T* 2014;39:776-92.

Waldman SA. Does potency predict clinical efficacy? Illustration through an antihistamine model. *Ann Allergy Asthma Immunol* 2002;89:7-11.
