# Supplementary material for: Ranking of antiseizure medications in a panel of focal seizure models predicts their comparative efficacy in clinical add‐on trials in drug‐resistant focal epilepsy
Source: Epilepsia. 2026 Mar 28;67(7):3719–37. doi: 10.1002/epi.70210 (PMC13360943; doi:10.1002/epi.70210)
Supplement: Supplementary file 3 — Figure S5. [file EPI-67-3719-s001.pdf]

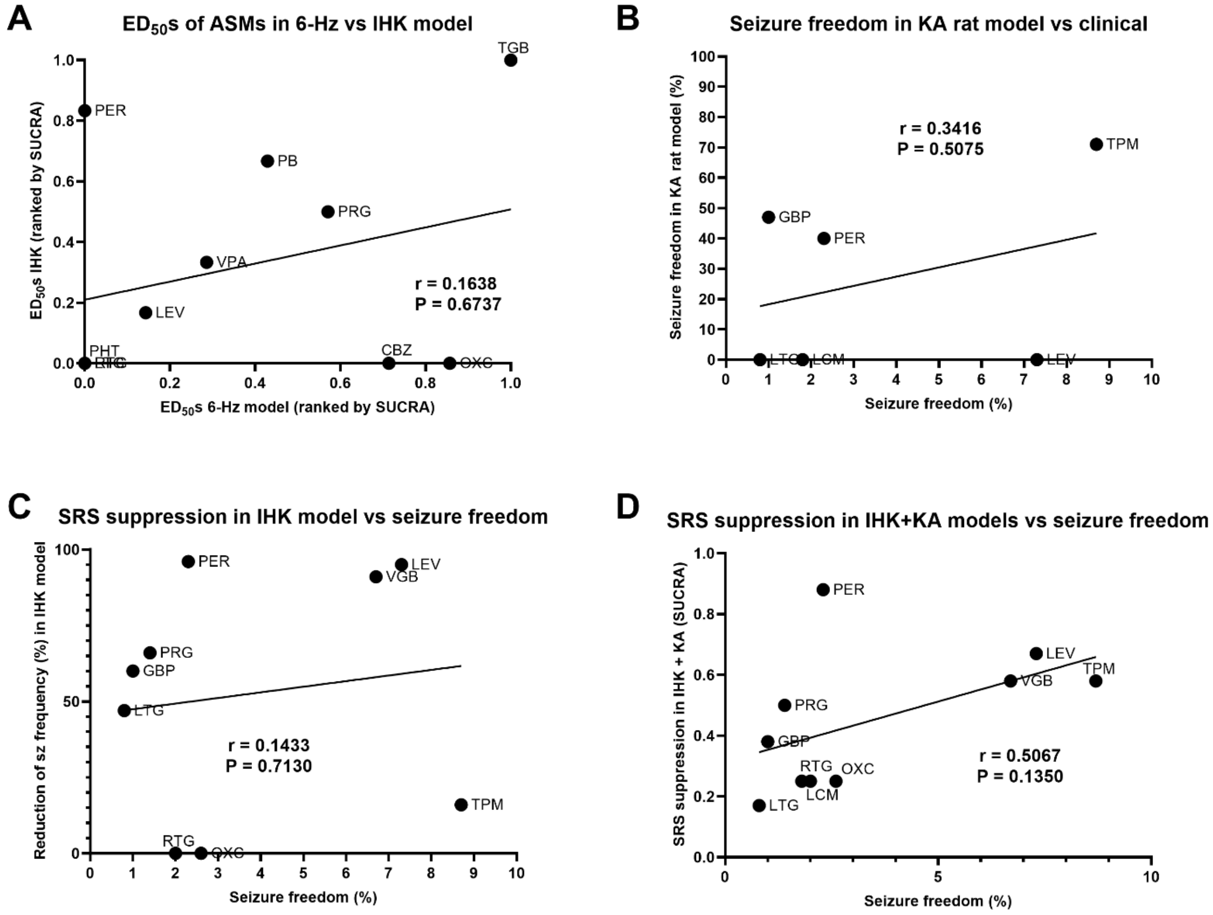

Fig. S5

Correlation analyses with potencies and efficacies of antiseizure medications (ASMs) in rodent models of chronic focal epilepsy with spontaneous recurrent seizures (SRS). The correlation coefficient r and the P value of r were determined by the method of Pearson and are indicated in each graph. A: Correlation analysis between the ranks of 11 ASMs in the 6-Hz model and the intrahippocampal kainate (IHK) model in mice. Ranking was based on ED_{50}s and performed by SUCRA (see Methods). Note that ED_{50}s are differently calculated by dose-response experiments in the two models. While ED_{50} in the 6-Hz model is the dose protecting 50% of mice from focal seizures, the ED_{50} in the IHK model represents the dose reducing focal seizure frequency by 50% in a group of epileptic mice (Löscher and White, 2023). B: Correlation between maximal seizure freedom determined in the systemic (i.p.) kainate (KA)

rat model and seizure freedom rates determined in randomized controlled add-on clinical trials (see Table 2). C: Correlation between maximal reduction of SRS frequency in the IHK model and clinical efficacy. D: Correlation between combined ranks for maximal SRS suppression in the IHK and systemic KA model and clinical efficacy.
