# Supplementary material for: Ranking of antiseizure medications in a panel of focal seizure models predicts their comparative efficacy in clinical add‐on trials in drug‐resistant focal epilepsy
Source: Epilepsia. 2026 Mar 28;67(7):3719–37. doi: 10.1002/epi.70210 (PMC13360943; doi:10.1002/epi.70210)
Supplement: Supplementary file 4 — Table S1. [file EPI-67-3719-s002.pdf]

Table S1. References for the preclinical antiseizure potencies of the 23 antiseizure medications (ASMs) shown in Table 1.

Drug	References
**First-generation ASMs**	
Phenytoin	Löscher et al., 1986; Turski et al., 1987; Lothman et al., 1988; Sofia et al., 1993; Guignet et al., 2020; Bosco et al., 2023
Carbamazepine	Löscher et al., 1986; Turski et al., 1987; Lothman et al., 1988; Sofia et al., 1993; Guignet et al., 2020; Bosco et al., 2023
Phenobarbital	Löscher et al., 1986; Lothman et al., 1988; Sofia et al., 1993; Guignet et al., 2020; Bosco et al., 2023
Primidone	Collins and Horlington, 1969; Gladding et al., 1985; Löscher et al., 1986; Lothman et al., 1988; Löscher and Nolting, 1991
Valproate	Löscher et al., 1986; Lothman et al., 1988; Sofia et al., 1993; Guignet et al., 2020; Koneval et al., 2020; Bosco et al., 2023
**Second- and third-generation ASMs**	
**1. SV2A modulators**	
Levetiracetam	Barton et al., 2001; Higgins et al., 2010; Rowley and White, 2010; Löscher et al., 2016; Leclercq et al., 2020; Bosco et al., 2023
Brivaracetam	Bialer et al., 2009; Matagne et al. 2008; Löscher et al., 2016; Leclercq et al., 2020; Bosco et al., 2023
**2. Sodium channel modulators**	
Oxcarbazepine	Borowicz et al., 2004; Clinckers et al., 2005; Booker et al., 2015; Bosco et al., 2023; PANAChE
Eslicarbazepine acetate	Potschka et al., 2014; Booker et al., 2015; Soares-da-Silva et al., 2015; Metcalf et al., 2017
Lamotrigine	Dalby and Nielsen, 1997; Guignet et al., 2020; Bosco et al., 2023
Lacosamide	Stöhr et al., 2007; Higgins et al., 2010; Nirwan et al., 2018; Guignet et al., 2020
**3. Calcium channel modulators**	
Gabapentin	Dalby and Nielsen, 1997; Metcalf et al., 2017; Bosco et al., 2023; PANAChE
Pregabalin	Vartanian et al., 2006; Huang et al., 2013; Luszczki et al., 2020
**4. GABAergic drugs**	
Vigabatrin	Löscher et al., 1989; Dalby and Nielsen, 1997; de Freitas, 2010; PANAChE; Florek-Luszczki et al., 2014
Tiagabine	Morimoto et al., 1996; Dalby et al., 1997; Barton et al., 2001; Salat et al., 2015; Metcalf et al., 2017; PANAChE
**5. Glutamate (AMPA) receptor antagonists**	
Perampanel	Hanada et al., 2011; Löscher and White, 2023
**6. ASMs with multiple mechanisms**	
Topiramate	Wauquier and Zhou, 1996; Zgrajka et al., 2010; Metcalf et al., 2017; Guignet et al., 2020; Bosco et al., 2023
Zonisamide	Kamai et al., 1981; Nakamura et al., 1994; Hamada et al., 2001; Shannon et al., 2005; Löscher and White, 2023
Rufinamide	White et al., 2008; Metcalf et al., 2017
Cenobamate	Bialer et al., 2013; Melnick et al., 2023; Leo et al., 2024; Melnick et al., 2025
Carisbamate	Klein et al., 2007; Bialer et al., 2009; Löscher et al., 2021

Felbamate	Sofia et al., 1993; Wlaz and Löscher, 1997; Guignet et al., 2020; Bosco et al., 2023
Retigabine (ezogabine)	Metcalf et al., 2017; Löscher et al., 2021; Bosco et al., 2023

References

Barton ME, Klein BD, Wolf HH, White HS. Pharmacological characterization of the 6 Hz psychomotor seizure model of partial epilepsy. *Epilepsy Res* 2001;47:217-28.

Bialer M, Johannessen SI, Levy RH, Perucca E, Tomson T, White HS. Progress report on new antiepileptic drugs: a summary of the Ninth Eilat Conference (EILAT IX). *Epilepsy Res* 2009;83:1-43.

Bialer M, Johannessen SI, Levy RH, Perucca E, Tomson T, White HS. Progress report on new antiepileptic drugs: a summary of the Eleventh Eilat Conference (EILAT XI). *Epilepsy Res* 2013;103:2-30.

Booker SA, Pires N, Cobb S, Soares-da-Silva P, Vida I. Carbamazepine and oxcarbazepine, but not eslicarbazepine, enhance excitatory synaptic transmission onto hippocampal CA1 pyramidal cells through an antagonist action at adenosine A1 receptors. *Neuropharmacology* 2015;93:103-15.

Borowicz KK, Luszczki JJ, Czuczwar SJ. SIB 1893, a selective mGluR5 receptor antagonist, potentiates the anticonvulsant activity of oxcarbazepine against amygdala-kindled convulsions in rats. *Pol J Pharmacol* 2004;56:459-64.

Bosco F, Guarnieri L, Leo A, Tallarico M, Gallelli L, Rania V *et al.* Audiogenic epileptic DBA/2 mice strain as a model of genetic reflex seizures and SUDEP. *Front Neurol* 2023;14:1223074.

Clinckers R, Smolders I, Meurs A, Ebinger G, Michotte Y. Hippocampal dopamine and serotonin elevations as pharmacodynamic markers for the anticonvulsant efficacy of oxcarbazepine and 10,11-dihydro-10-hydroxycarbamazepine. *Neurosci Lett* 2005;390:48-53.

Collins AJ, Horlington M. A sequential screning test based on the running component of audiogenic seizures in mice, including reference compound PD50 values. *Br J Pharmacol* 1969;37:140-50.

Dalby NO, Nielsen EB. Comparison of the preclinical anticonvulsant profiles of tiagabine, lamotrigine, gabapentin and vigabatrin. *Epilepsy Res* 1997;28:63-72.

de Freitas RM. Vigabatrine increases superoxide dismutase activity in striatum of rat after pilocarpine-induced seizures. *Arch Clin Psychiatr* 2010;37:36-40.

Florek-Luszczki M, Wlaz A, Luszczki JJ. Interactions of levetiracetam with carbamazepine, phenytoin, topiramate and vigabatrin in the mouse 6Hz psychomotor seizure model - a type II isobolographic analysis. *Eur J Pharmacol* 2014;723:410-8.

Gladding GD, Kupferberg HJ, Swinyard EA. Antiepileptic drug development program. In: Frey H-H, Janz D, eds. *Antiepileptic drugs.* Berlin: Springer, 1985:341-50.

Guignet M, Campbell A, White HS. Cenobamate (XCOPRI®): Can preclinical and clinical evidence provide insight into its mechanism of action? *Epilepsia* 2020;61:2329-39.

Hamada K, Song HK, Ishida S, Yagi K, Seino M. Contrasting effects of zonisamide and acetazolamide on amygdaloid kindling in rats. *Epilepsia* 2001;42:1379-86.

Hanada T, Hashizume Y, Tokuhara N, Takenaka O, Kohmura N, Ogasawara A *et al.*

Perampanel: a novel, orally active, noncompetitive AMPA-receptor antagonist that reduces seizure activity in rodent models of epilepsy. *Epilepsia* 2011;52:1331-40.

Higgins GA, Breysse N, Undzys E, Derksen DR, Jeffrey M, Scott BW *et al.* Comparative study of five antiepileptic drugs on a translational cognitive measure in the rat: relationship to antiepileptic property. *Psychopharmacology (Berl)* 2010;207:513-27.

Huang CW, Lai MC, Cheng JT, Tsai JJ, Huang CC, Wu SN. Pregabalin attenuates excitotoxicity in diabetes. *PLoS One* 2013;8:e65154.

Kamei C, Oka M, Masuda Y, Yoshida K, Shimizu M. Effects of 3-sulfamoylmethyl-1,2-benzisoxazole (AD-810) and some antiepileptics on the kindled seizures in the neocortex, hippocampus and amygdala in rats. *Arch Int Pharmacodyn Ther* 1981;249:164-76.

Klein BD, Smith MD, White HS. The novel neuromodulator carisbamate delays the acquisition of rat amygdala kindling and maintains acute antiepileptic activity when evaluated in postkindled rats. *American Epilepsy Society Annual Meeting Abstracts Online* 2007;Abstract number : 3.322.

Koneval Z, Knox KM, Memon A, Zierath DK, White HS, Barker-Haliski M. Antiseizure drug efficacy and tolerability in established and novel drug discovery seizure models in outbred vs inbred mice. *Epilepsia* 2020;61:2022-34.

Leclercq K, Matagne A, Provins L, Klitgaard H, Kaminski RM. Pharmacological profile of the antiepileptic drug candidate padsevonil - characterization in rodent seizure and epilepsy models. *J Pharmacol Exp Ther* 2020;372:11-20.

Leo A, Bosco F, Guarnieri L, De Sarro C, Rania V, Gallelli L *et al.* Cenobamate enhances the anticonvulsant effect of other antiseizure medications in the DBA/2 mouse model of reflex epilepsy. *Eur J Pharmacol* 2024;962:176222.

Lothman EW, Salerno RA, Perlin JB, Kaiser DL. Screening and characterization of antiepileptic drugs with rapidly recurring hippocampal seizures in rats. *Epilepsy Res* 1988;2:367-79.

Löscher W, Jäckel R, Czuczwar SJ. Is amygdala kindling in rats a model for drug-resistant partial epilepsy? *Exp Neurol* 1986;93:211-26.

Löscher W, Jäckel R, Müller F. Anticonvulsant and proconvulsant effects of inhibitors of GABA degradation in the amygdala-kindling model. *Eur J Pharmacol* 1989;163:1-14.

Löscher W, Nolting B. The role of technical, biological and pharmacological factors in the laboratory evaluation of anticonvulsant drugs. IV. Protective indices. *Epilepsy Res* 1991;9:1-10.

Löscher W, Gillard M, Sands ZA, Kaminski RM, Klitgaard H. Synaptic Vesicle Glycoprotein 2A Ligands in the Treatment of Epilepsy and Beyond. *CNS Drugs* 2016;30:1055-77.

Löscher W, Sills GJ, White HS. The ups and downs of alkyl-carbamates in epilepsy therapy: How does cenobamate differ? *Epilepsia* 2021;62:596-614.

Löscher W, White HS. Animal Models of Drug-Resistant Epilepsy as Tools for Deciphering the Cellular and Molecular Mechanisms of Pharmacoresistance and Discovering More Effective Treatments. *Cells* 2023;12:1233.

Luszczki JJ, Panasiuk A, Zagaja M, Karwan S, Bojar H, Plewa Z *et al.* Polygonogram and isobolographic analysis of interactions between various novel antiepileptic drugs in the 6-Hz corneal stimulation-induced seizure model in mice. *PLoS One* 2020;15:e0234070.

Matagne A, Margineanu DG, Kenda B, Michel P, Klitgaard H. Anti-convulsive and anti-

epileptic properties of brivaracetam (ucb 34714), a high-affinity ligand for the synaptic vesicle protein, SV2A. *Br J Pharmacol* 2008;154:1662-71.

Melnick SM, Shin Y, Glenn KJ. Anticonvulsant effects of cenobamate in chemically and electrically induced seizure models in rodents. *Heliyon* 2023;9:e18920.

Melnick SM, Misra SN, Kamin M, Ferrari L, Glenn KJ. Potential of cenobamate as a broad-spectrum antiseizure medication. *Expert Opin Pharmacother* 2025;26:1177-89.

Metcalf CS, West PJ, Thomson KE, Edwards SF, Smith MD, White HS *et al.* Development and pharmacologic characterization of the rat 6 Hz model of partial seizures. *Epilepsia* 2017;58:1073-84.

Morimoto K, Sato H, Yamamoto Y, Watanabe T, Suwaki H. Antiepileptic effects of tiagabine, a selective GABA uptake inhibitor, in the rat kindling model of temporal lobe epilepsy. *Epilepsia* 1997;38:966-74.

Nakamura J, Tamura S, Kanda T, Ishii A, Ishihara K, Serikawa T *et al.* Inhibition by topiramate of seizures in spontaneously epileptic rats and DBA/2 mice. *Eur J Pharmacol* 1994;254:83-9.

Nirwan N, Siraj F, Vohora D. Inverted-U response of lacosamide on pilocarpine-induced status epilepticus and oxidative stress in C57BL/6 mice is independent of hippocampal collapsin response mediator protein-2. *Epilepsy Res* 2018;145:93-101.

Potschka H, Soerensen J, Pekcec A, Loureiro A, Soares-da-Silva P. Effect of eslicarbazepine acetate in the corneal kindling progression and the amygdala kindling model of temporal lobe epilepsy. *Epilepsy Res* 2014;108:212-22.

Rowley NM, White HS. Comparative anticonvulsant efficacy in the corneal kindled mouse model of partial epilepsy: Correlation with other seizure and epilepsy models. *Epilepsy Res* 2010;92:163-9.

Salat K, Podkowa A, Kowalczyk P, Kulig K, Dziubina A, Filipek B *et al.* Anticonvulsant active inhibitor of GABA transporter subtype 1, tiagabine, with activity in mouse models of anxiety, pain and depression. *Pharmacol Rep* 2015;67:465-72.

Shannon HE, Eberle EL, Peters SC. Comparison of the effects of anticonvulsant drugs with diverse mechanisms of action in the formalin test in rats. *Neuropharmacology* 2005;48:1012-20.

Soares-da-Silva P, Pires N, Bonifácio MJ, Loureiro AI, Palma N, Wright LC. Eslicarbazepine acetate for the treatment of focal epilepsy: an update on its proposed mechanisms of action. *Pharmacol Res Perspect* 2015;3:e00124.

Sofia RD, Gordon R, Gels M, Diamantis W. Effects of felbamate and other anticonvulsant drugs in two models of status epilepticus in the rat. *Res Commun Chem Pathol Pharmacol* 1993;79:335-41.

Stöhr T, Kupferberg HJ, Stables JP, Choi D, Harris RH, Kohn H *et al.* Lacosamide, a novel anti-convulsant drug, shows efficacy with a wide safety margin in rodent models for epilepsy. *Epilepsy Res* 2007;74:147-54.

Turski WA, Cavalheiro EA, Coimbra C, da Penha BM, Ikonomidou-Turski C, Turski L. Only certain antiepileptic drugs prevent seizures induced by pilocarpine. *Brain Res* 1987;434:281-305.

Vartanian MG, Radulovic LL, Kinsora JJ, Serpa KA, Vergnes M, Bertram E *et al.* Activity profile of pregabalin in rodent models of epilepsy and ataxia. *Epilepsy Res* 2006;68:189-205.

Wauquier A, Zhou SD. Topiramate: A potent anticonvulsant in the amygdala- kindled rat. *Epilepsy Res* 1996;24:73-7.

White HS, Franklin MR, Kupferberg HJ, Schmutz M, Stables JP, Wolf HH. The anticonvulsant profile of rufinamide (CGP 33101) in rodent seizure models. *Epilepsia* 2008;49:1213-20.

Wlaz P, Löscher W. Anticonvulsant activity of felbamate in amygdala kindling model of temporal lobe epilepsy in rat. *Epilepsia* 1997;38:1167-72.

Zgrajka W, Nieoczym D, Czuczwar M, KiÅ› J, Brzana W, WlaÅº P *et al.* Evidences for pharmacokinetic interaction of riluzole and topiramate with pilocarpine in pilocarpine-induced seizures in rats. *Epilepsy Res* 2010;88:269-74.
